# Metatranscriptomics-based metabolic modeling of patient-specific urinary microbiome during infection

**DOI:** 10.1038/s41522-025-00823-6

**Published:** 2025-09-09

**Authors:** Jonathan Josephs-Spaulding, Hannah Clara Rettig, Johannes Zimmermann, Mariam Chkonia, Alexander Mischnik, Sören Franzenburg, Simon Graspeuntner, Jan Rupp, Christoph Kaleta

**Affiliations:** 1 Research Group Medical Systems Biology, University Hospital Schleswig-Holstein Campus Kiel, 24105 Kiel University, Kiel, Schleswig-Holstein Germany; 2 Institute of Medical Microbiology, University of Lübeck, 23538 Lübeck, Germany; 3 Infectious Disease Clinic, University Hospital Schleswig-Holstein Campus Lübeck, Lübeck, Germany; 4 Institute of Clinical Molecular Biology, University Hospital Schleswig-Holstein, Kiel University, Rosalind Franklin Strasse 12, 24105 Kiel, Germany; 5 German Center for Infection Research (DZIF), Partner Site Hamburg-Lübeck-Borstel-Riems, Lübeck, Germany

**Keywords:** Microbiome, Clinical microbiology

## Abstract

Urinary tract infections (UTIs) are among the most common bacterial infections and are increasingly complicated by multidrug resistance (MDR). While *Escherichia coli* is frequently implicated, the contribution of broader microbial communities remains less understood. Here, we integrate metatranscriptomic sequencing with genome-scale metabolic modeling to characterize active metabolic functions of patient-specific urinary microbiomes during acute UTI. We analyzed urine samples from 19 female patients with confirmed uropathogenic E. coli (UPEC) infections, reconstructing personalized community models constrained by gene expression and simulated in a virtual urine environment. This systems biology approach revealed marked inter-patient variability in microbial composition, transcriptional activity, and metabolic behavior. We identified distinct virulence strategies, metabolic cross-feeding, and a modulatory role for *Lactobacillus* species. Comparisons between transcript-constrained and unconstrained models showed that integrating gene expression narrows flux variability and enhances biological relevance. These findings highlight the metabolic heterogeneity of UTI-associated microbiota and point to microbiome-informed diagnostic and therapeutic strategies for managing MDR infections.

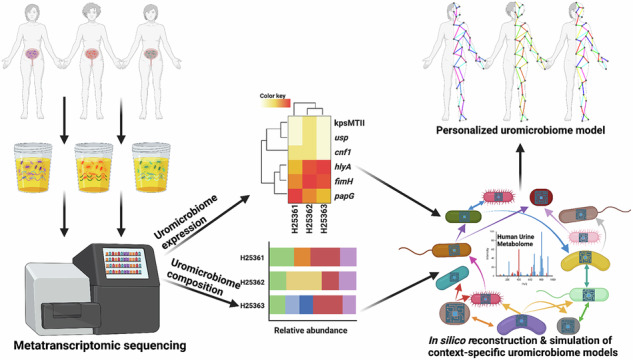

## Introduction

Urinary tract infections (UTIs) persist as one of the most common global health problems, with significant annual cases and healthcare costs reported worldwide^1^. Specifically, uncomplicated UTIs are not associated with anatomical abnormalities, but frequently affect women and may result in recurrent, chronic infections^2^. UTIs are diagnosed by the presence of uropathogenic *E. coli (*UPEC*)*, a bacterium found in approximately 80% of UTI cases^1^. Even with identifying a primary causative factor, the growing issue of multi-drug resistant (MDR) UTIs has impeded effective treatment; present antibiotic treatments have become increasingly ineffective and led to the elevation of UTIs from common infections to intricate medical challenges^3,4^. As a result, there is an urgent need for a detailed understanding of MDR UTI-associated microbial communities and the functional consequences of their metabolic crosstalk to develop novel therapeutic approaches for UTI treatment.

Scrutinizing microbial communities through shotgun metagenomics (sequencing of DNA from microbial communities) is routinely applied to investigate human diseases, but fewer studies aim to leverage metatranscriptomes (sequencing of RNA from microbial communities) across human niches. The main disadvantage is that metatranscriptomics is limited by low resolution in distinguishing species-level organisms from one another, as compared to metagenomics. However, metatranscriptomics offers the unique advantage that it can identify both microbial taxa and active transcripts, which can be used to describe microbial community responses to human hosts^5^. For example, metatranscriptomics has been used to investigate the vaginal microbiome during vaginosis to study *G. vaginalis* and how this microbe circumvents stress from the antibiotic metronidazole by expressing genes for DNA repair^6^. While RNA-seq alone has been utilized to investigate how uropathogenic *E. coli* responds in human urine and activates virulence mechanisms^7^, RNA-seq focuses only on species-specific responses. At the same time, metatranscriptomics can investigate the gene response from entire human-associated microbial communities.

To expand the utility of sequencing human microbiomes through mechanistic studies of community interactions and phenotypes, the application of genome-scale metabolic models (GEMs) is becoming widely popular. GEMs are mathematical reconstructions of the metabolic network of a species and provide a consolidated computational view into organismal physiology^8^. An increasingly common and robust approach to investigating microbial response to specific conditions or contexts is the application of GEMs constrained by OMICs data (transcriptomics, proteomics, or metabolomics) to effectively model microbial response to their environment^9,10^. While the AGORA2 database has provided a resource of 7203 gut-derived GEMs, which can be used to model drug transformation or bioremediation in a human niche accurately, context-specific GEMs have also been applied to study whole-body metabolism to simulate the interaction between hosts and microbes^11,12^. Recently, a reproducible and flexible framework has been developed to combine metatranscriptomics with GEMs to improve the functional understanding of microbial communities^13^.

Here, we explore UTI pathology and microbial community physiology by integrating metatranscriptomics with metabolic modeling to evaluate microbial functions from clinical isolates. We analyzed 19 UTI patient samples and assessed their respective microbial diversity and gene expression to construct GEMs for uropathogen communities to predict metabolism in these human-associated ecosystems. Simulations of microbial growth and cross-talk were enhanced by applying a generic in silico urine medium based on the comprehensive Human Urine Metabolome database^14,15^, aiding in reconstructing patient-specific uromicrobiomes. Our findings offer insights into the variable microbial responses in UTIs, supporting the development of personalized treatments that rely on metabolic reprogramming, rather than antibiotic treatments.

## Results

### UTI pathogenic microbiome composition and diversity vary across patients

**Fig. 1:**
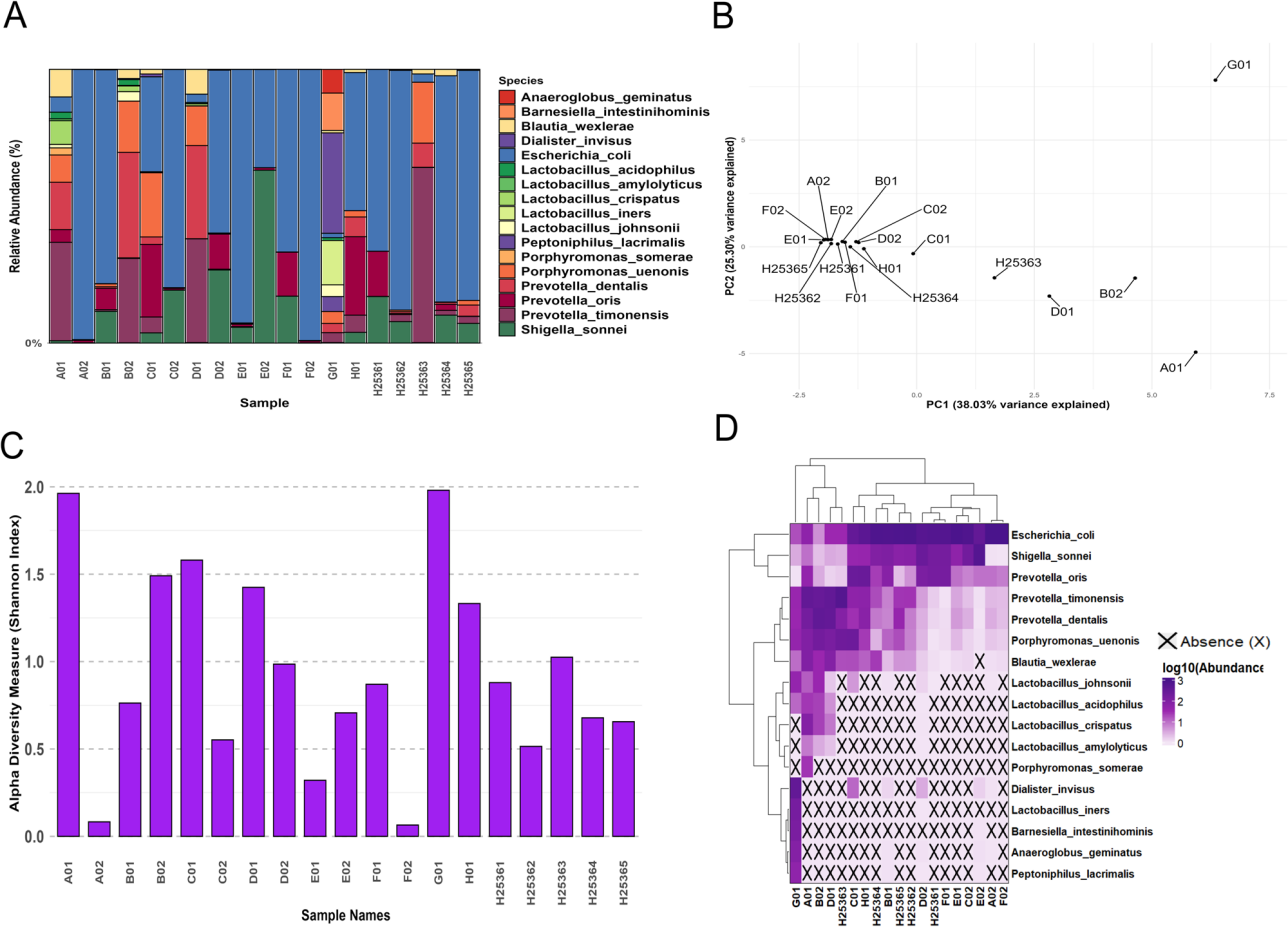
Diversity and composition of UTI microbiomes across patients. **A** Species-level relative abundance profiles across samples including uropathogens and non-urogenital genera such as Anaeroglobus, Barnesiella, Escherichia/Shigella, and Lactobacillus. **B** Principal Component Analysis (PCA) of species composition across patients. Notable outliers include samples A01, B02, and G01, all of these patients are linked to diverse Lactobacillus profiles. **C** Shannon alpha diversity indices across samples. **D** Heatmap of predicted species-level abundances across patients to inform species selection for microbial community modeling.

Our analysis revealed diverse UTI bacterial communities among patients, indicating inter-patient biodiversity variation in terms of composition and abundance (Fig. 1). Figure 1A presents predicted species-level relative abundance and a range of identified microbial taxa that define a microbial signature for each patient. Variable abundance of microbial groups were observed, including genera such as *Anaeroglobus, Barnesiella, Blautia, Dialister, Escherichia/Shigella, Lactobacillus, Peptoniphilus, Porphyromonas, and Prevotella* (Supplementary Fig. S2). Interestingly, while this analysis identified genera known to be potential urinary tract pathogens, other taxa are known to be human pathogens that are generally associated with the gut, oral, and vaginal-associated environments; these pathogens may have roles in urinary tract colonization and warrant further investigation^16–21^. Surprisingly, *Lactobacillus taxa* were prevalent, with patients having anywhere between 0-4 different species per sample. A meaningful abundance and diversity of *Lactobacillus taxa* were observed in samples A01, B02, and G01; thereby separating our cohort into two distinct groups of patients.

The impact of the abundance of these probiotic taxa can be distinguished in the Principal Component Analysis (PCA) of species composition which underlies that diversity in the studied uromicrobiome’s varied ecological landscape, with PC1 and PC2 explaining 38.03% and 25.30% of the variance, respectively (Fig. 1B). Shannon’s Alpha Diversity analysis reveals a simplified and narrow diversity range from 0.064 to 1.962, this is to be expected from an *E. coli* associated UTI. However patients with UTI communities containing *Lactobacillus* communities have increased diversity (Fig. 1C). To guide the downstream selection of species for microbial community modeling, we generated a relative abundance matrix of all taxa detected across the UTI patient cohort and performed hierarchical clustering (Fig. 1D). This analysis revealed co-occurrence patterns: *Prevotella* spp. (including *P. timonensis, P. dentalis, and P. oris*) clustered closely and were often enriched in tandem with *Enterobacteriaceae* (*E. coli* and *S. sonnei*). Notably, *Lactobacillus* species, including *L. crispatus and L. iners*, formed their own cluster and were largely absent in samples dominated by *E. coli*. The presence or absence of these core taxa informed model reconstruction.

### UPEC UTI89 virulence strategies vary across UTI patients

**Fig. 2:**
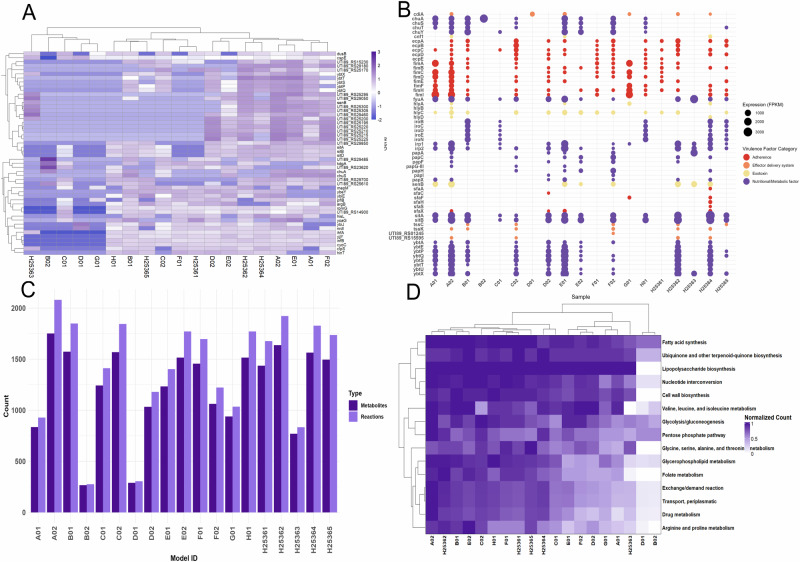
Virulence strategies and metabolic adaptation of UPEC UTI89 across patients. **A** Expression profiles of the top 50 most abundant microbially active transcripts that map to UPEC UTI89 across patients reveal consistent expression. **B** Virulence factors identified via VFDB showed consistently high expression of adhesion-related genes and iron uptake genes, suggesting conserved pathogenic adaptations. **C** Context-specific metabolic models reconstructed from transcripts mapped to UPEC UTI89 show wide variation in metabolic diversity. **D** Subsystem activity analysis shows variable expression in key metabolic pathways(arginine/proline metabolism, glycolysis/gluconeogenesis, peptide metabolism, and BCAA metabolism).

Gene expression data were mapped to the UPEC UTI89 reference genome to extract FPKM values, focusing on this strain as the primary cause of ~80% of UTIs^1^. The top 50 expressed genes across patient samples revealed both conserved and variable transcriptional patterns (Fig. 2A). Notably, *ssrA was* consistently the most abundant transcript while other frequently expressed genes include *rnpB, cspA*, and *ssrS*. Interestingly, several highly expressed UTI89 locus tags (approximately 20 out of the top 50 expressed genes) with unknown functions were also identified, highlighting the need for annotation of these genes and investigating their roles in UTI recurrence.

To explore pathogenic mechanisms, we annotated gene expression profiles using the Virulence Factor Database (VFDB)^22^ to reveal clinically relevant virulence traits that were unique across patients (Fig. 2B). Adhesion genes such as *fimA* and *fimI*, essential for epithelial colonization^23^, were expressed in multiple samples. Iron acquisition genes (*chuY*, *chuS*, and *iroN)* which are key to nutrient scavenging and metabolic adaptation, were also commonly expressed^24–26^. These variable expression patterns underscore UPEC’s flexible virulence strategies and its ability to adapt to diverse host environments, likely contributing to different clinical manifestations.

We next reconstructed context-specific metabolic models for patient-derived UPEC UTI89 strains as part of our microbiome community modeling workflow. While some gene expression profiles were conserved across samples, mapping expression data onto metabolic networks revealed distinct differences between taxonomically similar strains (Fig. 2C). Reaction and metabolite counts varied considerably across patient-specific UTI89 models, with some strains exhibiting expansive networks containing over 2000 reactions and 1800 metabolites, while others such as sample B02 had fewer than 300, suggesting a more constrained metabolic capacity.

We further analyzed these models to identify patterns in subsystem activity (Fig. 2D). For example, ‘arginine and proline metabolism’ was highly active in sample A02 (normalized activity = 0.882), but inactive in B02 and D01. Similarly, the pathways ‘drug metabolism’ and ‘glycolysis/gluconeogenesis’ were enriched in patients A02 and C02, but reduced in D01. ‘nucleotide interconversion’ was elevated in F02 and H01, but minimal in H25363 and H5365. The ‘pentose phosphate pathway’, was most active in A02 and largely inactive in D01 and B02. These findings emphasize the limitations of relying solely on gene expression data and highlight the importance of integrating context-specific metabolic modeling. Together, these results suggest that UPEC’s metabolic adaptability plays a central role in its pathogenicity and may inform patient-specific therapeutic strategies.

We summed a curated gene set of transcripts that were mapped to metabolic subsystems for each patient and compared them to flux predictions from a UTI89-specific GEM, using Pearson correlation to quantify alignment (Supplementary Figs. S3, S4). While most samples showed strong concordance in core pathways, sample B02 was a notable outlier, exhibiting uniformly low predicted fluxes despite robust transcript mapping, primarily to uncharacterized locus tags. This discrepancy may reflect expression of poorly annotated *E. coli* genes driving novel metabolic functions, or result from technical limitations in genome annotation or model constraints, warranting further investigation.

### Differential microbiome metabolic activity in context-specific and non-context-specific models

**Fig. 3:**
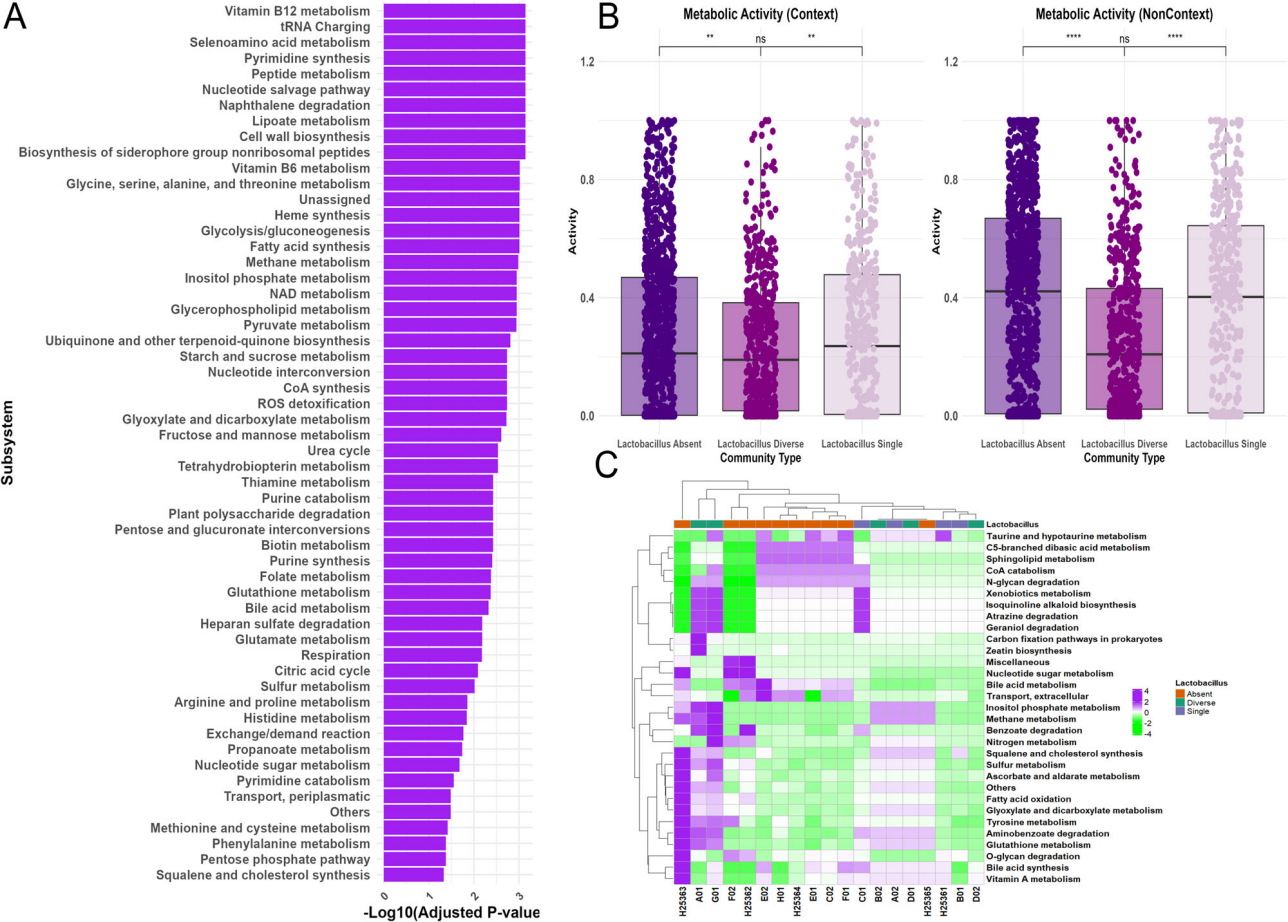
Differential metabolic activity in context-specific vs. non-context uromicrobiome models. **A** Context-specific microbiome models showed significant changes in metabolic subsystem activity compared to non-context-specific conditions. **B** Metabolic activity differed across manually defined Lactobacillus community types: Lactobacillus Diverse (*n* = 5), Lactobacillus Single (*n* = 4), and Lactobacillus Absent (*n* = 10). **C** A heatmap of log fold changes in metabolic subsystem activity identified highly variable pathways in comparison to non-context-specific models.

We generated microbiome-wide metabolic subsystem abundance data for each patient to compare two simulation approaches: context-specific and non-context-specific microbiome community models, using BacArena (Fig. 3A). Both simulations focused on microbial communities associated with patient-specific UTIs.Non-context-specific simulations were based on consistent *gapseq* GEMs that were gap-filled with an in silico urine medium. In contrast context-specific models incorporated additional sample-specific constraints through metatranscriptomics data, refining the same models using gene expression profiles. These simulations aimed to evaluate whether context-specific data could highlight biologically relevant metabolic patterns that are potentially related to pathogenic processes in UTIs. Paired *t* tests identified significant subsystem differences under context-specific conditions, particularly in ‘biosynthesis of siderophore group nonribosomal peptides’ (adjusted *p*-value = 7.24 × 10^−4^), ‘fatty acid synthesis’ (adjusted *p*-value = 9.77 × 10^−4^), and ‘glycolysis/gluconeogenesis’ (adjusted *p*-value = 9.77 × 10⁻⁴). Additional subsystems, including ‘cell wall biosynthesis’ and ‘peptide metabolism’ (both adjusted *p*-value = 7.24 × 10^−4^), were also significantly altered.

PCA (Supplementary Fig. S5A) summed components explained 30.30% of the variance and highlighted reduced variability in context-specific models compared to non-context-specific models which contain more outliers and extraneous metabolic reactions. We observed a decreased total metabolic flux in context-specific models (Supplementary Fig. S5B), suggesting a stronger focus on biologically relevant pathways. Analysis of Euclidean distance analysis presented sample-specific differences in metabolic flux (Supplementary Fig. S5C), further supporting that applying context-specific modeling can capture adaptive metabolic changes related to environmental context by improving biological relevance in microbiome metabolic modeling. We further scrutinized context-specific model viability through flux variability analysis (FVA). FVA supported reduction of context-specific GEM solution space through reduction of the model’s solution space and elimination of artificially induced flux constraints (i.e +/−, 500, 750, 1000, etc. flux boundaries). FVA also identified reduced mean flux variability in context-specific GEMs and a higher proportion of reactions with zero variability, indicating a narrower solution space and tighter metabolic regulation driven by transcriptomic constraints (Supplementary Fig. S6).

Given that several patients harbored between 0–5 active *Lactobacillus* strains, we compared metabolic fluxes across manually defined *Lactobacillus* communities under both simulation types (Fig. 3B). Samples were categorized into three groups: *Lactobacillus* Diverse (≥3 species, *n* = 5), *Lactobacillus* Absent (no species, *n* = 10), and *Lactobacillus* Single (1 species, *n* = 4). Pairwise comparisons revealed that the *Lactobacillus* Absent group exhibited the highest mean metabolic activity under both conditions, followed by *Lactobacillus* Single, and then *Lactobacillus* Diverse. When applying an ANOVA test with Tukey’s post-hoc test, a significant difference between the Absent and Diverse groups was observed (*p* < 0.001), and between Single and Diverse (*p* < 0.001), in both simulation types. These findings suggest that the presence and diversity of *Lactobacillus* strains substantially influence community-level metabolic activity in UTI microbiomes. Additionally, across all groups, context-specific models consistently exhibited lower metabolic activity compared to non-context-specific models (*p* < 0.001), underscoring how transcriptomic integration constraints and refines functional predictions.

We assessed individual community metabolic flux data by calculating fold-changes between our two computational reconstruction conditions for individual patients (Fig. 3C). Log fold changes were first computed by comparing metabolic subsystem activity between context-specific and non-context specific conditions for each patient. Metabolic subsystems with low variance across all studied patient samples were filtered out to retain subsystems with the highest variability (log fold change > 0.30), to present the most significant differences observed when applying context-specific modeling.

### Metabolic flux analysis of production and consumption processes in microbial communities

**Fig. 4:**
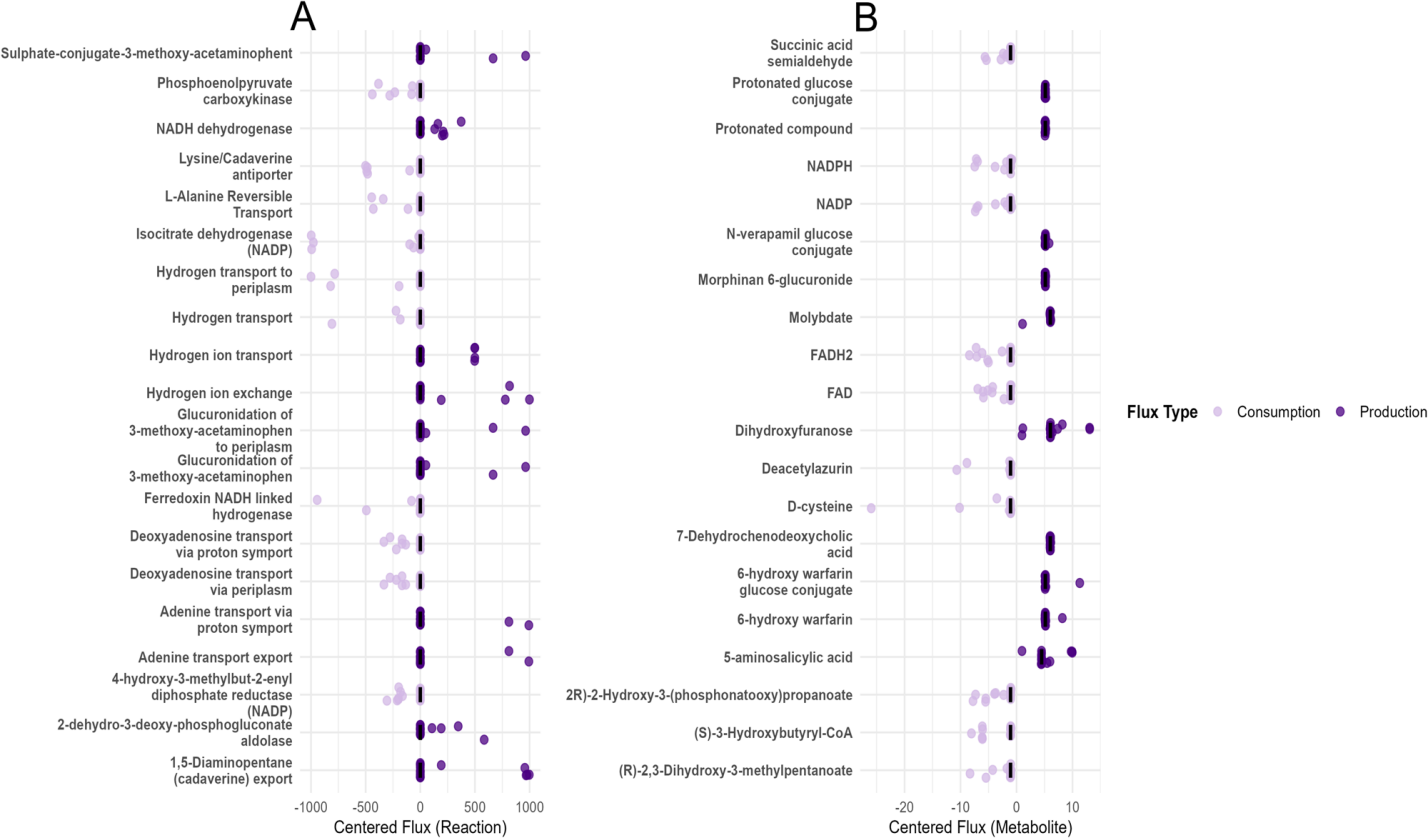
Key metabolic fluxes associated with microbial interactions in UTI communities. **A** Flux analysis identified key metabolic reactions based on high mean flux and *t* test significance. Major production pathways included ferredoxin activity, cholate transport, and glycerol-3-phosphate acyltransferase. Top consumption pathways included hypoxanthine and cholanate exchange, and 5-dehydro-D-gluconate reductase. **B** Metabolite-level analysis revealed significant production of fermentation products such as CO_2_, ammonium, ethanol, and L-lactate along with D-alanine, deoxyadenosine diphosphate, and guanosine.

We reconstructed microbiome-wide community models for each patient using metatranscriptomic sequencing data and generated two datasets per patient to compare metabolic differences between two experimental approaches: context-specific and non-context-specific microbiome community models, using BacArena simulations. We then analyzed metabolic fluxes within microbial communities, focusing on both positive (production) and negative (consumption) reactions (Fig. 4). Reactions with significant deviations in metabolic flux were identified, top reactions were selected based on both the magnitude of their mean flux values and statistical significance after normalizing each flux by the patient’s microbiome community's mean biomass flux.

Among the positive flux reactions, context-specific models exhibited high exports of 2-dehydro-3-deoxy-phosphogluconate aldolase, 1,5 diaminopentane (cadaverine), hydrogen ion exchange, and proton driven adenine import/export, and glucuronide conjugate export of 3-methyl acetaminophen. These significant fluxes point to active detoxification and amine secretion processes that likely support community survival in the urinary tract. Conversely, the top consumption reactions in context-specific models included isocitrate dehydrogenase (NADP⁺), hydrogen uptake, lysine/cadaverine antiport, ferredoxin-linked hydrogenase, and PEP carboxykinase, all are related to TCA intermediates, proton motive forces, and nucleoside salvage (Fig. 4A).

The highest production metabolites in context-specific models were dihydroxy furanose, 7-dehydro chenodeoxycholic acid, molybdate, glucose–warfarin conjugates, and 5-aminosalicylic acid, all representing sugar derivatives, bile-acid conjugation, and stress-metabolite handling. Top consumption included D-cysteine, FADH₂, (S)-3-hydroxybutyryl-CoA, 3-phosphoglycerate, and NADPH, highlighting uropathogens optimization of amino-acid scavenging, redox cofactor cycling, and glycolytic flux activity (Fig. 4B).

### Interplay of metabolite cross-feeding in UTI microbial communities reveals potential pathogenic strategies

**Fig. 5:**
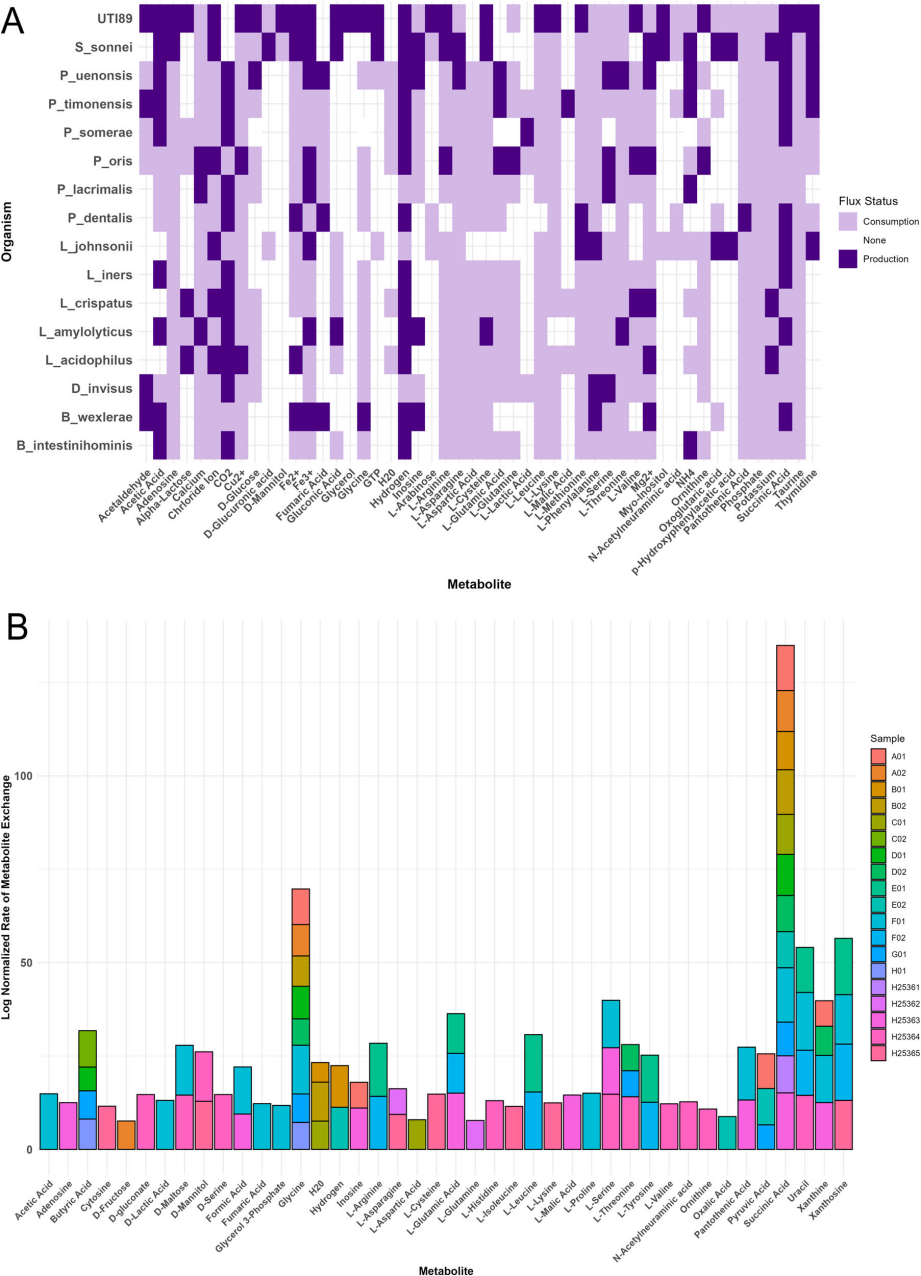
Metabolic cross-feeding and metabolite exchange in patient-specific microbiome. **A** Cross-feeding interactions were assessed by analyzing extracellular exchange (EX_) metabolite production and consumption rates across microbial species in each patient’s microbiome model. Key contributors to exchange were identified based on flux magnitude. **B** Log-normalized exchange rates revealed patient-specific variability. Uracil and L-leucine showed elevated exchange rates F01, F02, and E01, while succinic acid and glycine were consistently exchanged across multiple patients.

Metabolic cross-feeding across members of context-specific patient-specific microbiomes were examined by analyzing metabolite flux data, focusing on production (positive flux) and consumption (negative flux) rates across different organisms within each community. Our analysis centered on metabolic exchange reactions, which are boundary reactions that enable metabolite import or export between organisms and their environment, (i.e nutrient uptake, waste secretion, and interspecies interactions). By calculating the average fluxes for each metabolite-organism pair, we identified trends in the most actively exchanged metabolites (Fig. 5A).

Within the community model simulation, several *Lactobacillus* species contributed substantially to the production of alpha-lactose, a metabolite involved in pH regulation in both gut and vaginal microbiomes. Species such as *L. rhamnosus* and *L. reuteri* were also involved in fermenting carbohydrates into short-chain fatty acids (SCFAs), compounds essential for maintaining gut health. In contrast, GEMs mapped to *UTI89* were associated with the consumption of various SCFAs, including acetate, suggesting a strategy of nutrient acquisition and adaptation within the urinary tract. A particularly interesting finding was the production of ornithine by UTI89 and its consumption by other uromicrobiome members. Ornithine has previously been linked to enhanced fitness and possible competitive advantage for uropathogens. These interactions suggest a potential cooperative or exploitative dynamic between *Lactobacillus* species and UTI89, with the former producing metabolites that may facilitate pathogenic activity. While direct acetate cross-feeding from *Lactobacillus spp* and UTI89 was not observed, it is plausible that acetate released into the extracellular environment is subsequently taken up by UTI89.

Further analysis revealed additional trends in cross-fed metabolites. Probiotic microbiota such as *L. amylolyticus* were found to produce L-threonine, hydrogen, and succinic acid, some of the most commonly exchanged metabolites across communities. In contrast, *D. invisus* and *P. lacrimalis* showed consistent consumption of phosphate, L-glutamic acid, and D-glucose, indicating a strong metabolic dependence on these compounds. Other taxa, including *L. iners* and *Bifidiobacter intestinihominis* produced and consumed taurine and glycine. These findings suggest a complex web of metabolic interdependencies within the uromicrobiome, where some microbes act primarily as producers and others as consumers. Such cross-feeding interactions likely influence both microbial community stability and pathogenic potential.

To assess broader patterns, we examined the log-normalized rate of metabolite exchange across the entire cohort of context-specific microbiome community models with the aim to identify cohort-wide trends and patient-specific signatures of metabolite exchanges (Fig. 5B). We found substantial variability in exchange fluxes, with certain metabolites displaying notably high activity in specific samples. For example, uracil and L-leucine showed elevated exchange in patients F01, F02, and E01, suggesting pathogens in these patients rely on metabolic pathways associated with these compounds. Succinic acid and glycine, both key metabolic intermediates, were consistently exchanged across numerous samples, reinforcing their role in UTI community metabolism and, in the case of succinate, its link to pro-inflammatory signaling^27^. Other metabolites, such as xanthosine, L-glutamic acid, and acetic acid, showed more sample-specific exchange patterns. Simulated metabolite exchange results for each patient can be found in Supplementary Table S5. Together, these findings illustrate the individualized nature of microbial metabolism in the urinary tract and underscore the importance of cross-feeding in shaping microbial community structure and pathogenic potential.

## Discussion

The outdated belief that the urinary tract is sterile has been recently refuted, with evidence now supporting the existence of a unique microbiome within the urinary tract, even in the absence of infections^28,29^. Our study extends metatranscriptomics with systems biology tools to explore the urinary microbiome of women experiencing UTIs to reveal a network of microbial compositions, functions, and interactions. Contrary to the notion that UTIs are caused by single pathogens, our findings, and those of others, suggest a communal and synergistic bacterial interplay^30,31^. By integrating metatranscriptomics with GEM reconstruction and applying metabolic constraints through the Human Urine Metabolome Database, we generated personalized microbiome models for individual patients^14^. This approach improves our understanding of the metabolic dynamics within the UTI microbiome by emphasizing microbial diversity and complexity in the urinary tract of UTI patients. Recognizing these intricate microbial interactions is essential to devise more effective, patient-specific treatments.

The diversity of the uromicrobiome among UTI patients highlights the need for personalized therapeutic strategies, challenging the generalized nature of standard treatment protocols. This diversity, which includes uropathogenic, oral-associated, gut-associated, and probiotic genera, suggests that UTIs may arise from complex microbial interactions rather than from a single etiological agent. Such variability can influence immune responses, infection susceptibility, and treatment outcomes, with contributing factors such as genetics, diet, age, and antibiotic usage all affecting disease manifestation^32,33^. While the *Lactobacillus* genus is known to confer protective benefits and prevent pathogen colonization in the urinary tract; the role of these microbes in *E. coli* diagnosed UTIs remains unclear. We employed the Shannon index to measure patient-specific uromicrobiome diversity, an approach applicable to other body sites as well^15,34^. However, traditional diversity metrics fail to capture the influence of low-abundance species, which can affect community dynamics and disease progression. These overlooked minor taxa, may be important to understand urinary health and disease, and require more nuanced analytical approaches^35,36^. Our findings also identified gut-associated microbes such as *E. coli and S. sonnei* in the urinary tract, suggesting possible microbial migration between gastrointestinal and urogenital systems. While this supports the hypothesis of cross-site microbial transfer, this observation must be interpreted cautiously in the absence of matched fecal sample data^33,37–39^. Although our filtering pipeline minimized environmental contamination by cross-validating metatranscriptomic taxa with supplemental 16S rDNA data and manually verifying taxa through literature review, we cannot completely rule out the possibility of contamination during sample collection or processing. Additionally, we observed minor discrepancies in sequencing data between Illumina MiSeq and NovaSeq platforms due to differences in sequencing depths and bias, a known challenge in cross-platform microbiome comparisons^40^.

Analysis of the top 50 expressed genes in UPEC UTI89 across patient samples revealed both conserved and variable transcriptional patterns. Frequent detection of *cspA*, and *ssrS* suggests a conserved stress response and transcriptional regulation within UPEC UTI89^41,42^. This homeostatic expression of specific genes may indicate a mechanism for UPEC survival and function in host urinary environments. In contrast, variability in the expression of other genes highlights UPECs ability to adapt its transcriptional profile in response to host-specific pressures^43^. The high expression of UTI89 locus tags, or genes lacking assigned gene names, especially in sample B02, suggest potential biological and clinical significance. Characterization of these loci, through efforts such as the *E. coli* ‘y-ome’, may reveal novel molecular functions, mechanisms, or biomarkers relevant to immune responses and disease progression^44,45^. Notably, sample B02’s low flux despite high locus-tag transcript counts underscores the need for targeted functional characterization of these unannotated regions to determine whether they represent novel metabolic activities or technical artifacts of sequencing and model mapping. Mapping patient-specific gene expression to UTI89 further revealed diverse virulence strategies, with differential expression of virulence genes among UTI patients, complicating infection management due to distinct pathogenic mechanisms^7,46^. Genes such as *fimA* and *fimI*, associated with cell adherence, were highly expressed in certain samples, indicating enhanced host cell invasion and infection persistence^47^. Other genes like *chuY* and *chuS (*heme utilization), and *iroN* and *fepA (*iron acquisition), underline UPEC’s strategies to acquire essential nutrients in the iron-limited urinary tract, crucial to their virulence and survival^24–26^. This multifaceted virulence profile, combining adherence, invasion, and nutritional acquisition, may contribute to infection severity and resistance to treatment^48^.

Context-specific metabolic reconstructions represent considerable variability in UPEC UTI89’s metabolic networks, across patient samples. Metatranscriptome data indicated a wide range of gene activity, with some functionally active taxa displaying extensive metabolic networks involving over 2000 reactions and 1800 metabolites, while others demonstrated more limited profiles. This suggests that taxa with conserved gene expression may employ different metabolic strategies^49–51^. Such variation may reflect adaptive responses enabling UPEC to optimize metabolism for specific host environments. However, differences in RNA extraction efficiency across patient samples may also contribute to the observed variability. Subsystem analysis revealed further patient-specific differences in activity, particularly in pathways such as ‘arginine and proline metabolism,’ ‘drug metabolism,’ ‘glycolysis/gluconeogenesis’, and ‘pentose phosphate pathway’. These differences, enriched in metatranscriptomic-derived taxa from certain patients (e.g., A02 and C02) but limited in others (e.g., D01), likely influence UPEC’s virulence and fitness^50,52–54^.

Comparison of context-specific and non-context-specific microbiome community models revealed notable differences in pathway activity, highlighting the utility of environmental context in GEMs^55–57^. Non-context-specific models consistently predicted higher activity than context-specific ones, underscoring the need for applying contextual model constraints to reflect realistic biological processes. Subsystems such as ‘biosynthesis of siderophore group nonribosomal peptides,’ ‘fatty acid synthesis,’ and ‘glycolysis/gluconeogenesis’ presented substantial changes in metabolic flux activity under context-specific conditions, reflecting adaptation to environmental metabolites to trigger virulence mechanisms^50,58,59^. The reduced flux variability in context-specific models, as shown by PCA and flux analyses, supports their biological relevance by focusing on essential metabolic pathways^56^. These results suggest that tailoring models to environmental conditions improves the resolution and interpretability of microbiome-wide metabolic behavior. Differences in activity across *Lactobacillus* community types highlight the influence of microbial composition on metabolism. While *E. coli* remains the primary pathogen driving infection, the presence and gene expression of co-inhabiting Lactobacillus *species* may influence infection dynamics and patient outcomes. Including *Lactobacillus* in our in silico microbiome community model allowed us to capture microbial interactions that are likely important in UTI pathophysiology. The *Lactobacillus* *Absent* group showed the highest activity, followed by the *Lactobacillus Single* and *Lactobacillus Diverse* groups, under both modeling conditions. Statistical analyses revealed significant differences between *Lactobacillus Diverse* and the other two groups, suggesting that higher *Lactobacillus* diversity may correspond with lower simulated metabolic activity. These findings suggest a complex relationship between *Lactobacillus* diversity and uropathogen-associated metabolism, warranting further investigation^60^. Fold-change analysis of subsystem activity highlighted pathways that may support pathogen adhesion and adaptation in host environments affected by dysfunction in other organ systems^61–63^. Conversely, sphingolipid metabolism showed minimal activity in some samples, but elevated activity in others, suggesting niche-specific metabolic constraints possibly linked to biofilm formation^64^. These results reinforce the advantages of integrating expression data into metabolic modeling to identify microbe-specific adaptations.

In our metabolite and reaction-level FBA, the highest predicted export fluxes were for sugar and stress-related metabolites: dihydroxy furanose (a sugar derivative), 7-dehydro chenodeoxycholic acid (a bile acid conjugate), molybdate, glucose–warfarin conjugate, and 5-aminosalicylic acid. These findings point to enhanced carbohydrate processing and bile-acid modification pathways, along with detoxification of stress compounds^65^. Notably, elevated fluxes for both deoxyadenosine and adenine transport suggest an increased demand for purine biosynthesis, which is critical for nucleic acid metabolism and cellular growth^66,67^. By contrast, the largest uptake fluxes were for D-cysteine, FADH₂, (S)-3-hydroxybutyryl-CoA, 3-phosphoglycerate, and NADPH, consistent with active amino-acid scavenging and continued glycolytic/redox cofactor cycling to balance energy needs. This pattern is consistent with known microbial community metabolism: for example, gut microbes are well-known to transform bile acids into diverse conjugates, and microbial populations commonly exchange metabolites (organic acids, amino acids, sugars, etc.) through cross-feeding and waste detoxification^68^. These results show that context-specific modeling constraints narrow the model’s solution space and channels flux through biologically relevant detoxification, cross-feeding, and energy-balancing pathways, supporting that our key predictions derive from patient-specific gene expression rather than arbitrary medium assumptions. Finally, cross-feeding analysis within patient-specific microbiomes demonstrated interspecies exchange of key metabolites. *Lactobacillus* species (i.e *L. rhamnosus* and *L. reuteri)*, were major producers of alpha-lactose and SCFAs like acetate, which help modulate pH and support probiotic balance. However, the uptake of these metabolites by uropathogens may facilitate recurrent microbial infections^69,70^. Ornithine production by UTI89, with uptake by other community members, may reflect a competitive advantage in fitness^53,71^. Other contributions by *L. amylolyticus*, including L-threonine and succinic acid, further illustrate probiotic roles in supporting pathogen metabolism^51,72^. These findings suggest how individual species support interspecies uropathogenic communities through metabolite exchange across host environments. Variation in exchange rates across patient-specific microbiome models reflects the personalized nature of microbial metabolism. High metabolic turnover of uracil and L-leucine exchange in patients F01, F02, and E01 suggest uropathogens leverage enriched pathways to salvage pyrimidines and uptake amino acids, promoting anti-persister characteristics^73,74^. Succinic acid consistently showed high exchange rates, indicating a central metabolic role and possible pro-inflammatory activity^27,51^. These context-specific exchanges between microbes and host-shaped metabolic environments underscore the importance of personalized approaches in understanding microbial community and host health.

While exploratory in nature, our findings offer the potential for translational applications for managing MDR UTIs across diagnostics, therapeutics, and microbiome-based interventions. The integration of metatranscriptomics with GEMs allowed us to identify metabolic signatures of infection that are tied to taxa-related flux patterns, which could be developed into diagnostic biomarkers or therapeutic targets^13,74^. Several urinary metabolites, including acetate, trimethylamine, and agmatine, have been previously shown to have a high sensitivity and specificity for predicting UTI presence and pathogen identity in metabolomics-based diagnostic studies^14,28,31,75,76^. Patient-specific GEMs may also support personalized treatment by predicting pathogen nutrient dependencies and identifying optimal intervention points, including targeted antibiotics, metabolic inhibitors, or probiotics^54,55,77^. With regards to novel treatments, intravaginal administration of *Lactobacillus crispatus* significantly reduced UTI recurrence in clinical trials^78^. Oral D-mannose, which competitively inhibits *E. coli* adhesion, has shown efficacy comparable to antibiotics in preventing recurrence^79^; these options and others can be further investigated through constraint-based GEMs. Additionally, disrupting microbial cross-feeding of metal-based cofactors that were observed in our GEMs such as iron, may impair pathogenic survival or biofilm formation^80,81^. Overall, our exploratory in silico analysis at the systems-level supports the development of future microbiome and metabolic-informed clinical tools for precision UTI care.

Our metatranscriptomics-based modeling of the UTI microbiome offers valuable insight into metabolic pathways and cross-feeding interactions relevant to UTIs. These findings lay the groundwork for follow-up studies to pursue targeted interventions, such as dietary supplements to disrupt pathogenic activity. However, the presented models have limitations. Microbial phenotypes are shaped by individual and environmental factors, including diet, health status, microbial compositions, pH, and nutrient availability, which are not fully captured in our framework. As we work toward a more precise definition of the functional uromicrobiome, future models will integrate patient-specific dietary and metabolic data, improving simulations and guiding clinical decision-making. This demonstrates the utility of metatranscriptomics to uncover dynamic host-microbiome interactions and point towards a future of personalized UTI treatments based on microbiome compositions.

## Methods

### Clinical assessment and study criteria

Samples to reconstruct and microbiome community models were derived from a study cohort of patients sampled from November 2018 to August 2019 at the University Hospital Schleswig-Holstein and the University of Lübeck. The study was approved by the institutional review board (“Ethikkommission”) of the University of Lübeck on February 1st 2018 with the application number AZ18-009. The study comprises female patients who were 18 years or older at the time of sampling, who presented acute symptoms of uncomplicated UTIs with confirmed evidence of culturable uropathogenic *E. coli* from self-sampled mid-stream urine. Mid-stream urine was taken according to the recommendations of the German Association of Urology (DGU)^82^ and taken immediately before antibiotic therapy. UTIs with *E. coli* were confirmed by incubating fresh urine on chromID CPS Elite agar plates (Biomérieux SA, Marcy-l‘Étoile, France). If *E. coli*-like colonies were identified on the plates, they were further classified with a MALDI Biotyper (Bruker Daltonik GmbH, Bremen, Germany). Samples were included into the study only if results were clearly interpretable as *E. coli* in two independent measurements.

While additional phenotypic information was not included to further describe patients due to the small cohort size, patients were included or excluded from this study based on previously described recommendations by the DGU. Specifically, patients were excluded if they presented physical malformations or comorbidities indicative of a complicated UTI (i.e anatomical urogenital malformations (urethral stricture, bladder stones or tumors), previous urinary tract surgery, neurological or other diseases affecting bladder emptying, prior pelvis radiation/radiotherapy, cytostatic treatment within the past year or during the study, immunosuppression, type 1 or 2 diabetes mellitus, pregnancy, recent use of vaginal probiotic therapies or vaginal spermicides, and participation in clinical studies potentially affecting urinary or renal function within 30 days before or during the study) as recommended by the DGU^82^.

### RNA and DNA isolation for metatranscriptomic and 16s rDNA sequencing

In total, 19 total RNA (i.e Metatranscriptomics) and 16s rDNA sequences were obtained from patients with a confirmed UTI. Negative isolation controls were included in each extraction and processed identically for each sample to monitor reagent contamination. Total RNA was extracted from urine samples using the MICROBEnrich and MICROBExpress mRNA enrichment kits (Ambion, Thermo Fisher Scientific) to reduce human RNA contamination and enrich for bacterial RNA. Purification was performed with the TruSeq Stranded Total RNA kit (Illumina) to enable shotgun metatranscriptomic sequencing and functional profiling of the urinary microbiome. Sequencing was conducted at the Competence Centre for Genomic Analysis (CCGA) at Christian-Albrecht University, Kiel. Initially, samples H25361–H25365 were sequenced on an Illumina MiSeq platform (2×75 bp paired-end reads). As additional resources became available, 14 more samples were sequenced on an Illumina NovaSeq 6000 platform with 2×50 bp paired-end reads). We performed concurrent 16 s rDNA sequencing from 19 matched samples of urine using the AllPrep DNA/RNA Mini Kit (Qiagen GmbH, Hilden, Germany), following the manufacturer’s protocol for targeted amplification of the V3/V4 regions to establish a positive control for our urine metatranscriptome sequencing protocol^83^. A positive control was selected to provide additional independent, unbiased confirmation of microbial taxa detected in metatranscriptomes through 16 s rDNA to validate true biological presence, rather than testing for environmental contamination of the urinary tract samples. DNA was stored at −20°C for further analysis. Partial 16S rDNA gene sequences were amplified using indexed primers targeting V3/V4^83,84^. Amplicons were pooled in equimolar amounts, purified with the MinElute Gel Extraction Kit (Qiagen), and quantified prior to library preparation^85^. Sequencing was performed on an Illumina MiSeq platform using the MiSeq Reagent Kit V3 (600 cycles). The PhiX library served as a positive control for sequencing accuracy, and all negative controls remained free of amplification. Sample details, including IDs and sources are provided in Supplementary Table S1. Rigorous quality controls were applied across all sequencing workflows. Negative isolation controls were processed alongside samples to monitor reagent purity and potential contamination. Only samples with clearly defined PCR amplicons proceeded to sequencing and analysis, while controls confirmed no detectable contamination. While all metatranscriptomic sequences had good quality for analysis, 4 16 s rDNA samples were excluded due to poor sequencing quality.

### Metatranscriptomics preprocessing

**Fig. 6:**
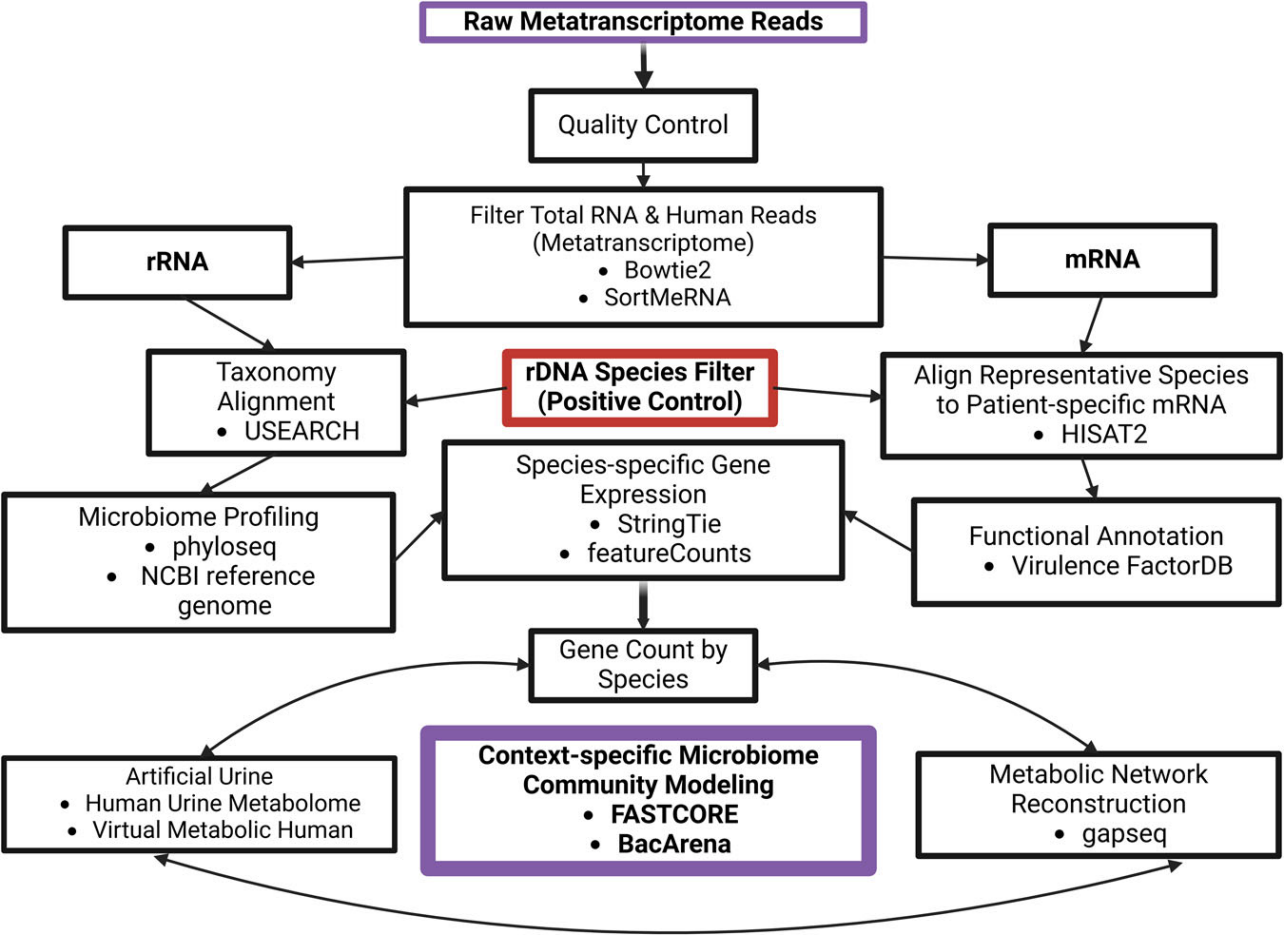
Workflow of reconstructing context-specific microbiome community models from metatranscriptomic data. Nineteen raw metatranscriptomic datasets were processed to generate patient-specific microbiome community models for investigating UTI-related metabolic interactions. After separating rRNA and mRNA fractions, two parallel pipelines were used to assess taxonomic and functional profiles, respectively, before integration for model reconstruction. Microbial rRNA reads were clustered into OTUs and aligned to microbial reference databases for taxonomic profiling, with species identities verified through complementary 16S rDNA sequencing. Reference genomes of identified species were retrieved NCBI, and metatranscriptome-derived mRNA reads were mapped to these genomes to infer gene expression Gene-level counts were integrated into genome-scale metabolic reconstructions to generate context-specific models reflective of each patient’s uromicrobiome activity. Finally, these models were introduced into a virtual simulation using a defined Human Urine Metabolome medium to explore cross-feeding and microbe–environment interactions.

An overview of the metatranscriptomics workflow, from raw RNA reads to reconstruction of context-specific microbiome community models, is shown in Fig. 6. Preprocessing begins with quality control (QC) of both total RNA and 16S rDNA sequencing data to ensure high-quality reads, separates mRNA from rRNA for metabolic modeling, and isolate microbial rDNA as a positive control to confirm the presence of microbial taxa identified by metatranscriptomics, enabling us to filter for true-positive uromicrobiome members and exclude potential contaminants. Sequence quality was assessed using FastQC (v0.11.8)^86^, identifying low-quality reads for removal. Adapters and low-quality sequences were trimmed using Cutadapt (v1.5)^87^ and PRINSEQ-lite (v0.20.4)^88^. Cutadapt trimmed adapters and low-quality bases from paired-end reads were removed if they were shorter than 1 bp or with quality scores below 10, with a minimum 5 bp overlap for adapter detection. Illumina and sample-specific adapter sequences were removed with PRINSEQ to retain a minimum length of 1 bp and an average quality score ≥ 10, allowing up to 5 ambiguous bases per read. Low-quality bases were trimmed from both ends, with up to 10 bases removed from the 5’ and 3’ ends.

To remove human RNA contamination, total RNA reads were aligned to the GRCh38 human reference genome (GCF_000001405.26) using Bowtie2 (v2.5.4) with default alignment parameters. Aligned reads, representing human sequences, were discarded, and non-human reads being retained for downstream metatranscriptomic analysis. SortMeRNA (v4.3.7)^89^ was then used to classify retained reads, employing seven indexed databases, including Silva-Bac16s and Silva-Bac23s databases. Reads not mapping to rRNA were assumed to be mRNA and used for functional analysis and context-specific metabolic model reconstruction. Mapping rates are provided in Supplementary Table S2. All pre-processed metatranscriptome-derived rRNA and mRNA in addition to our positive control 16 s rDNA were clustered with CD-HIT (v4.8.1)^90^ to reduce redundancy, using a 95% identity threshold and a word size of 5.

### Metatranscriptomics taxonomy and species filtering

To identify microbial taxa from metatranscriptome-derived rRNA and 16s rDNA data, paired-end reads were merged using ‘fastq_mergepairs’ in USEARCH(v12.0)^91^. Redundant sequences and singletons were removed with ‘fastx_uniques’ to generate a labeled, non-redundant dataset. Denoising and chimera filtering were performed using ‘unoise3’, clustering sequences into OTUs (OTUs) at 95% identity to eliminate errors and artifacts. A 95% bootstrap confidence threshold was applied to retain biologically relevant OTUs as a cross-reference to the literature. Microbial abundance was quantified with ‘usearch_global’ matching reads to OTUs at ≥ 95% identity. Taxonomic classification was performed with ‘sintax’ against the RDP 16S reference database, using a 0.95 confidence cutoff.

To distinguish genuine host-associated microbes from potential environmental contaminants in the metatranscriptomic rRNA, 16S rDNA sequencing was used as a biological positive control. Since 16S rDNA is derived from total microbial genomic DNA, it provides an orthogonal, DNA-based snapshot of microbial presence that is independent of transcriptional activity. Cross-referencing taxa identified in the metatranscriptomic rRNA data with those detected in the 16S rDNA dataset, we filtered out species likely representing sequencing noise, transient contaminants, or environmental organisms not stably colonizing the urinary tract. Only taxa consistently detected in both the metatranscriptomic rRNA and 16S rDNA datasets were retained for downstream analysis. To further validate biological relevance, each retained taxon was subjected to a manual literature review through a range of digital published libraries (i.e NCBI, PubMed, Google Scholar, clinical reports). Literature database searches are formatted to consistently use combinations of microbe-specific taxonomic nomenclature with keywords such as “clinical”, “urine”, “human microbiome”, and “infection”. Taxa with literature support for association with human-associated or urinary microbiota were kept, while those linked primarily to environmental niches (e.g., soil, water, plants) were excluded.

Post computational and manual filtering, clean mRNA and rRNA reads were categorized into transcriptional units (TUs) and OTUs, PCA was used to assess potential technical bias from sequencing platform differences (Supplementary Fig. S1). We computed PCA loadings on rRNA‑derived OTUs and mRNA‑derived TUs to investigate if platform‑driven features derived from technical bias in our metatranscriptome sequencing (MiSeq vs. NovaSeq). We additionally rarefied each sample to equal read depth, to diminish traces of sequencing‑platform clustering in PCA (Supplementary Fig. S1).

Phyloseq (v1.5)^92^ was used to assign taxonomy and estimate the across patient samples. A phyloseq object was generated by integrating OTU and taxonomy tables from USEARCH with curated sample metadata. OTUs were filtered out if present in fewer than two samples or with fewer than ten total reads. Relative abundance was calculated using the ‘transform_sample_counts’ function, which normalizes OTU counts by total reads per sample. Only taxa with at least one non-zero value above this threshold were retained for downstream analysis.

### Functional profiling and gene expression quantification for metabolic modeling

The remaining mRNA reads, separated from rRNA by SortMeRNA, were used for functional analysis and species-specific gene expression quantification to inform community metabolic modeling. HISAT2 (v2.1.0)^93^, SamTools (v1.16)^94^, and FeatureCounts (v2.0.3)^95^ were used sequentially for read alignment, processing, and gene quantification. Reference genomes and annotations (Supplementary Table S3) were retrieved from NCBI based on species identified from the previously described taxonomy filtering to build species-specific HISAT2 genome indexes; mRNA reads were aligned to their respective genome indexes. mRNA reads were aligned to their respective genome indexes, then converted, sorted and indexed as SAM and BAM files using SamTools. FeatureCounts were then used to assign aligned reads to annotated genes. StringTie^96^ was used to quantify gene expression and assemble species-level transcriptomes, generating FPKM-normalized expression values per gene. These values were compiled into a master abundance matrix for cohort-level comparisons. As *E. coli* was clinically confirmed as an inclusion factor in all samples, gene-level FPKM values were aligned against the *E. coli* UPEC UTI89 reference genome (GCF_000013265.1) to identify virulence-associated genes. Genes were annotated using the Virulence Factor Database (VFDB)^22^ and classified by functional relevance in UTI pathology: adherence, effector delivery systems, exotoxins, and nutritional/metabolic factors. Gene-level expression values were summed across curated enzyme gene sets for each metabolic subsystem in *E. coli* UTI89 and compared to predicted fluxes from a UTI89-specific genome-scale model. For each subsystem, we computed Pearson’s correlation between summed transcript abundance and model-predicted flux to visualize patient-level association between genes and simulated metabolic activity.

### In silico human urine medium

To improve the accuracy of context-specific microbial community models, we developed an in silico urine metabolic medium based on the Urine Metabolome Database^14^, which catalogs 445 metabolites identified via NMR and GC-MS. Of these, 140 metabolites were deemed usable for microbial metabolic exchange reactions, after cross-referencing the Virtual Metabolic Human (VMH) database^15^. VMH metabolites that are available to microbes (i.e ‘phosphate’ listed as ‘Human (FALSE)/Microbe(TRUE)’, maps to the exchange reaction ‘EX_pi(e)’) were retained. Metabolite concentrations from the Human Metabolome Database were converted to millimolar (mM) units, normalized using the databases average creatinine levels, and adjusted to reflect conjugate base forms of acids^97^. The final in silico urine medium for simulating our microbial community models is detailed in Supplementary Table S4.

### Reconstruction and preprocessing of context-specific metabolic models

Patient-specific GEMs were reconstructed with *gapseq* (v1.3.1) to simulate microbial community metabolism in silico^98^. Reference genomes corresponding to species identified from metatranscriptomics analysis Supplementary Table S3 were used to predict metabolic pathways, transport reactions, and metabolic networks. Draft models were refined using our custom in silico urine medium (Supplementary Table S4). The *gapseq ‘*find’ command was used with ‘-p all’ (all pathway databases) and ‘-m bacteria’ (bacteria genomes), using a BLAST bit-score threshold 200. Membrane transport reactions were identified using gapseq ‘find-transport’. Draft metabolic models were generated using the *gapseq ‘*draft’ command, to incorporate genome annotations, reactions, and transport systems, with predefined stoichiometric and thermodynamic constraints. Models were gap-filled and constrained with our urine medium with gene-reaction mappings to ensure metabolic feasibility within our simulated urine environment (Supplementary Table S4).

To construct context-specific GEMs, we integrated gene expression data (FPKM-normalized) with the draft GEMS. Expression values were mapped to genes within each model based on chromosomal coordinates and linked to metabolic reactions using the COBRA Toolbox (v2.45.2) in MATLAB^99^. Reaction expression was defined conservatively as the minimum expression level among associated genes using ‘mapExpressionToReactions’. The ‘fastcc’ algorithm^100^ was applied to remove thermodynamically infeasible reactions and establish consistent flux-capable networks. Core active reactions were then identified using FastCore, with reactions in the top 25% of expression values designated as “active”^101^. These were used to reconstruct final context-specific models for each patient sample. Flux balance analysis (FBA) was performed on the reconstructed models using ‘optimizeCbModel’ with the biomass objective functions (BOF) as the objective function to confirm if the model’s metabolic viability was consistent with the reconstructed networks.

### Simulation of microbiome-specific community models

Patient-specific community models were simulated in BacArena(v1.8.1)^102^, by placing reconstructed context-specific GEMs into a 100×100 grid arena seeded with a total of 1000 cells. This relative abundance is reflective of patient-specific taxonomic profiles and simulations included to explore community growth, simulated flux, and cross-feeding interactions. An in silico *u*rine medium (Supplementary Table S4), annotated with VMH-derived exchange reactions and flux bounds, was formatted for BacArena. We conducted two simulation types^1^: “non-context” simulations using GEMs gap-filled with this urine medium and made flux-consistent via *fastcc*, and^2^ “context-specific” simulations applying the same models further constrained by species-level FPKM normalized gene expression data. Each simulation ran for four one-hour iterations in triplicate. Outputs including species growth, cross-feeding interactions, and metabolic flux distributions (extracted via ‘findFeeding3’) were compared between conditions to reveal transcriptome-driven differences in community-level and species-specific metabolic behaviors.

### Analysis of community simulations

Metabolic subsystem abundance was predicted for each patient’s context-specific and non-context-specific microbiome community models using BacArena simulations. We simulated patient-specific microbial communities using both context-specific and non-context-specific models in BacArena to evaluate how transcriptomic constraints influence overall community metabolism. By comparing flux variability and interspecies interactions between the two conditions, we aimed to reveal whether context-specific modeling narrows metabolic flexibility and better reflects functionally relevant cross-feeding dynamics in urinary microbiomes. To assess the impact of context-specific metabolic modeling, we performed paired t-tests for each metabolic subsystem, followed by Benjamini-Hochberg correction (significance threshold: adjusted *p* < 0.05). Significant *p*-values were transformed to -log10(p) for downstream analysis. We compared metabolic flux distributions across conditions and community types to evaluate functional differences. We also computed log₂ fold-changes in subsystem activity between context and non-context models for each patient. Subsystems with low variance (<0.30) were filtered out to retain those with meaningful differences, and hierarchical clustering was performed using Euclidean distance. We analyzed both positive (production) and negative (consumption) fluxes across reactions and metabolites, based on BacArena-predicted flux values. One-sample t-tests were used to determine whether fluxes significantly deviated from zero. Top reactions for production and consumption were selected based on significance and magnitude of mean flux. To investigate cross-feeding, we analyzed microbial metabolite production and consumption rates across species, focusing on extracellular exchange (EX_) reactions. For each metabolite-organisms pair, average fluxes were calculated to identify the most actively exchanged metabolites to reveal metabolic dependencies within patient-specific communities.

Due to the observation of some *Lactobacillus* communities in UTI confirmed patients, samples were grouped into three manually defined microbial community types:*Lactobacillus Diverse* (≥3 species, *n* = 5), *Lactobacillus Absent* (no *Lactobacillus*, *n* = 10), and *Lactobacillus Single* (dominated by one species, *n* = 4). ANOVA with Tukey’s post-hoc tests was applied to assess statistical differences in metabolic activity among these groups.

## Supplementary information

Supplementary_Tables.

Uromicrobiome_Supp_Figures.

## Data Availability

All raw sequencing data generated in this study have been deposited in the NCBI Sequence Read Archive (SRA) under BioProject accession number PRJNA1091563. This includes metatranscriptomic and 16S rDNA datasets from all patient samples.

## Code availability

All code, processed data, and metadata necessary to reproduce the figures and findings reported in this manuscript are publicly available on GitHub (https://github.com/PoeticPremium6/UTI-Microbiome-Model).
