# Supplementary material for: Metatranscriptomics-based metabolic modeling of patient-specific urinary microbiome during infection
Source: NPJ Biofilms Microbiomes. 2025 Sep 9;11:183. doi: 10.1038/s41522-025-00823-6 (PMC12420794; doi:10.1038/s41522-025-00823-6)
Supplement: Supplementary file 1 — Supplementary_Tables. [file 41522_2025_823_MOESM1_ESM.pdf]

| |
|---|
| **Table S1: Sample names, barcodes, and adaptor sequences used in this study.** |
| **Table S2: Preprocessing/quality control, rRNA or mRNA metrics acquired from metatranscriptomes. Alignment rate of metatranscriptomes across various mRNA databases** |
| **Table S3: Microbial metabolic models that were reconstructed and used for simulating host-specific models. Relative abundance for simulation input for each species.** |
| **Table S4: In silico human urine medium used in this study** |
| **Table S5: Bidirectional simulated metabolic interactions between producer and consumer strains** |

| Name | RNA | DNA | Sequencing Technology |
|---|---|---|---|
| H25361 | TRUE | TRUE | Illumina MiSeq |
| H25362 | TRUE | TRUE | Illumina MiSeq |
| H25363 | TRUE | TRUE | Illumina MiSeq |
| H25364 | TRUE | TRUE | Illumina MiSeq |
| H25365 | TRUE | TRUE | Illumina MiSeq |
| A01 | TRUE | FALSE | Illumina NovaSeq |
| A02 | TRUE | TRUE | Illumina NovaSeq |
| B01 | TRUE | TRUE | Illumina NovaSeq |
| B02 | TRUE | FALSE | Illumina NovaSeq |
| C01 | TRUE | FALSE | Illumina NovaSeq |
| C02 | TRUE | TRUE | Illumina NovaSeq |
| D01 | TRUE | TRUE | Illumina NovaSeq |
| D02 | TRUE | TRUE | Illumina NovaSeq |
| E01 | TRUE | TRUE | Illumina NovaSeq |
| E02 | TRUE | TRUE | Illumina NovaSeq |
| F01 | TRUE | TRUE | Illumina NovaSeq |
| F02 | TRUE | FALSE | Illumina NovaSeq |
| G01 | TRUE | TRUE | Illumina NovaSeq |
| H01 | TRUE | TRUE | Illumina NovaSeq |

| | Total Raw Sequences | rRNA/tRNA Reads (%) | mRNA Reads (%) | Bac rRNA | Euk rRNA | tRNA |
|---|---|---|---|---|---|---|
| **H25361** | 1.40E+07 | 91.93% | 8.07% | 90.58% | 0.39% | 0.96% |
| **H25362** | 9.94E+06 | 91.20% | 8.80% | 71.23% | 19.70% | 0.27% |
| **H25363** | 1.09E+07 | 92.89% | 7.11% | 0.92% | 91.85% | 0.13% |
| **H25364** | 1.27E+07 | 77.05% | 22.95% | 54.29% | 22.38% | 0.38% |
| **H25365** | 1.20E+07 | 97.54% | 2.46% | 66.60% | 30.40% | 0.55% |
| **A01** | 5.19E+07 | 84.49% | 15.51% | 9.25% | 74.80% | 0.45% |
| **A02** | 7.78E+07 | 97.06% | 2.94% | 94.84% | 2.18% | 0.03% |
| **B01** | 6.21E+07 | 87.56% | 12.44% | 74.92% | 12.14% | 0.49% |
| **B02** | 4.62E+07 | 89.10% | 10.90% | 8.61% | 80.41% | 0.08% |
| **C01** | 1.06E+07 | 20.98% | 79.02% | 9.20% | 11.47% | 0.32% |
| **C02** | 6.99E+07 | 98.24% | 1.76% | 96.15% | 2.08% | 0.02% |
| **D01** | 7.35E+07 | 79.69% | 20.31% | 0.95% | 78.49% | 0.26% |
| **D02** | 6.81E+07 | 93.68% | 6.32% | 88.92% | 4.73% | 0.02% |
| **E01** | 7.05E+07 | 98.16% | 1.84% | 94.41% | 3.59% | 0.15% |
| **E02** | 2.32E+07 | 98.14% | 1.86% | 97.88% | 0.18% | 0.06% |
| **F01** | 6.93E+07 | 96.69% | 3.31% | 95.79% | 0.74% | 0.15% |
| **F02** | 7.35E+07 | 97.13% | 2.87% | 95.23% | 1.81% | 0.08% |
| **G01** | 5.57E+07 | 82.39% | 17.61% | 58.63% | 23.64% | 0.12% |
| **H01** | 5.63E+07 | 92.88% | 7.12% | 47.45% | 45.34% | 0.10% |

| Organism | Refseq ID | A01 | A02 | B01 | B02 | C01 | C02 | D01 | D02 | E01 | E02 | F01 | F02 | G01 | H01 | H25361 | H25362 | H25363 | H25364 | H25365 |
|---|---|---|---|---|---|---|---|---|---|---|---|---|---|---|---|---|---|---|---|---|
| Shigella sonnei | GCF_013374815. | 8.434609 | 0.089755 | 116.0754 | 1.044932 | 37.41497 | 193.3535 | 2.128375 | 267.0667 | 58.00335 | 631.8637 | 171.2596 | 0.034949 | 2.148785 | 39.15848 | 169.9436 | 78.34576 | 1.767084 | 102.1199 | 72.09488 |
| Prevotella timonensis | GCA_000455445 | 359.8767 | 1.346318 | 5.863144 | 308.255 | 57.82313 | 1.041775 | 378.7387 | 2.861759 | 2.402505 | 0.122979 | 0.07021 | 1.188271 | 36.08477 | 62.80713 | 0.612865 | 25.8344 | 639.8296 | 17.34471 | 25.09401 |
| Prevotella dentalis | GCF_000242335. | 172.7734 | 1.929722 | 5.417842 | 385.5799 | 27.21088 | 1.840469 | 340.2039 | 1.769367 | 3.260543 | 0.061489 | 0.193077 | 1.118373 | 33.41731 | 72.02088 | 0.371505 | 6.430745 | 88.42681 | 6.97177 | 40.55101 |
| Porphyromonas uenonis | GCF_000482365. | 99.76419 | 0.807791 | 10.9099 | 187.0428 | 234.6939 | 0.41671 | 144.3934 | 1.492422 | 1.37286 | 0.122979 | 0.21063 | 0.594136 | 43.86485 | 23.18796 | 0.059157 | 5.790108 | 222.2653 | 1.622228 | 17.05057 |
| Blautia wexlerae | GCF_025148125. | 100.6711 | 0.134632 | 1.187472 | 33.43783 | 17.0068 | 0.347258 | 90.62395 | 0.630818 | 0.257411 | 0 | 0.017552 | 0.244644 | 9.039715 | 11.5172 | 0.141977 | 3.652329 | 16.62995 | 23.4143 | 3.760322 |
| Barnesiella intestinihominis | GCF_000296465. | 0 | 0 | 0 | 0 | 0 | 0 | 0 | 0 | 0 | 0.061489 | 0 | 0 | 136.559 | 0 | 0 | 0 | 0 | 0 | 0 |
| Dialister invisus | GCF_000160055. | 0 | 0.089755 | 0.371085 | 0 | 10.20408 | 0 | 0 | 2.138626 | 0 | 0.491914 | 0 | 0 | 366.5531 | 0 | 0 | 0 | 0 | 0 | 0 |
| Lactobacillus iners | GCF_009556455. | 0 | 0 | 0 | 0 | 0 | 0 | 0 | 0.015386 | 0 | 0 | 0 | 0 | 162.048 | 0 | 0 | 0 | 0 | 0 | 0 |
| Anaeroglobus geminatus | GCF_000239275. | 0 | 0.044877 | 0.074217 | 0 | 0 | 0 | 0 | 0.107701 | 0 | 0.122979 | 0 | 0 | 86.54416 | 0 | 0 | 0 | 0 | 0 | 0 |
| Lactobacillus johnsonii | GCF_014058685. | 12.60657 | 0.044877 | 0.074217 | 34.48276 | 3.401361 | 0 | 0.784138 | 0.400031 | 0 | 0 | 0 | 0 | 42.8275 | 0 | 0.002366 | 0 | 0 | 0 | 0 |
| Lactobacillus crispatus | GCF_009769205. | 86.52276 | 0 | 0 | 20.89864 | 0 | 0 | 7.281282 | 0.030772 | 0 | 0 | 0 | 0 | 0 | 0 | 0 | 0 | 0 | 0 | 0 |
| Peptoniphilus lacrimalis | GCF_900454715. | 0 | 0 | 0.074217 | 0 | 0 | 0 | 0 | 0.015386 | 0 | 0.061489 | 0 | 0 | 54.46058 | 0 | 0 | 0 | 0 | 0 | 0 |
| Porphyromonas somerae | GCF_022845615. | 25.93869 | 0 | 0 | 0 | 0 | 0 | 0 | 0 | 0 | 0 | 0 | 0 | 0 | 0 | 0 | 0 | 0 | 0 | 0 |
| Prevotella oris | GCF_900637655. | 46.345 | 8.302293 | 78.37316 | 2.089864 | 265.3061 | 5.625586 | 0.448079 | 128.3022 | 8.451671 | 8.362541 | 160.7456 | 6.500542 | 0.074096 | 286.7015 | 164.8395 | 3.976129 | 0.677786 | 22.15634 | 1.108571 |
| Lactobacillus acidophilus | GCF_034298135. | 55.68656 | 987.21 | 781.5793 | 4.179728 | 346.9388 | 797.3747 | 30.35734 | 595.1381 | 926.2517 | 358.7284 | 667.5033 | 990.3191 | 16.37522 | 504.6069 | 664.0291 | 875.9705 | 30.40352 | 826.3707 | 840.3406 |
| Lactobacillus amylolyticus | GCF_003999355. | 24.39688 | 0 | 0 | 20.89864 | 0 | 0 | 4.03271 | 0.015386 | 0 | 0 | 0 | 0 | 10.00296 | 0 | 0 | 0 | 0 | 0 | 0 |
| | | 6.983494 | 0 | 0 | 2.089864 | 0 | 0 | 1.008177 | 0.015386 | 0 | 0 | 0 | 0 | 0 | 0 | 0 | 0 | 0 | 0 | 0 |

| cpd_ID | VMH (exchange reaction) | Common Name | Concentration (µM/mM creatinine) | |
|---|---|---|---|---|
| cpd00726 | EX_13dampp(e) | 1,3-Diaminopropane | 1.2 | |
| cpd01866 | EX_1mncam(e) | 1-Methylnicotinamide | 5.8 | |
| cpd03561 | EX_2hb(e) | 2-Hydroxybutyrate | 2.8 | |
| N/A | EX_CE4970(e) | 2-Methylbutyrylglycine | 2 | |
| cpd03320 | EX_3hphac(e) | 3-Hydroxyphenylacetate | 6.65 | |
| cpd00136 | EX_4hbz(e) | 4-Hydroxybenzoate | 2.05 | |
| cpd02305 | EX_34hpl(e) | 4-HYDROXYPHENYLLACTATE | 3.2 | |
| cpd00631 | EX_4pyrdx(e) | 4-Pyridoxate | 3.1 | |
| cpd00337 | EX_56dura(e) | 5, 6-Dihydrouracil | 3.8 | |
| cpd00071 | EX_acald(e) | Acetaldehyde | 2.6 | |
| cpd00029 | EX_ac(e) | Acetate | 13 | |
| pd00178 | EX_acetone(e) | Acetone | 3.9 | |
| cpd01682 | EX_acrn(e) | O-Acetylcarnitine | 2.4 | |
| cpd00128 | EX_ade(e) | Adenine | 2.9 | |
| cpd00182 | EX_adn(e) | Adenosine | 1.4 | |
| cpd01092 | EX_alltn(e) | Allantoin | 15.4 | |
| cpd00705 | EX_L2aadp(e) | L-2-Aminoadipate | 7.3 | |
| cpd00123 | EX_3mob(e) | 3-Methyl-2-Oxobutanoate | 2.05 | |
| cpd00417 | EX_abt(e) | L-Arabinitol | 31.8 | |
| cpd00059 | EX_ascb_L(e) | L-Ascorbate | 32.5 | |
| cpd00085 | EX_ala_B(e) | Beta-Alanine | 5.9 | |
| cpd00540 | EX_glyb(e) | Glycine Betaine | 11.5 | |
| cpd00211 | EX_but(e) | Butyrate | 1.6 | |

| | | | | |
|---|---|---|---|---|
| cpd00310 | EX_carn(e) | Carnosine | 3.35 | |
| cpd00098 | EX_chol(e) | Choline | 3.5 | |
| cpd00333 | EX_cinnm(e) | trans-Cinnamate | 1.6 | |
| cpd00331 | EX_HC00342(e) | Cis-Aconitate | 18.1 | |
| cpd00137 | EX_cit(e) | Citrate | 203 | |
| cpd00250 | EX_creat(e) | Creatine | 46 | |
| cpd00585 | EX_crtn(e) | Creatinine | 1 | |
| cpd00307 | EX_csn(e) | Cytosine | 4.1 | |
| cpd00108 | EX_gal(e) | D-Galactose | 7.6 | |
| cpd00027 | EX_glc_D(e) | D-Glucose | 36.55 | |
| cpd00673 | EX_56dthm(e) | Dihydrothymine | 2.4 | |
| cpd00756 | EX_dmgly(e) | Dimethylglycine | 4.4 | |
| cpd00306 | EX_xylt(e) | Xylitol | 0.0004261820521 | |
| cpd26831 | EX_xyl_D(e) | D-Xylose | 20 | |
| cpd00363 | EX_etoh(e) | Ethanol | 3.1 | |
| cpd00162 | EX_etha(e) | Aminoethanol | 37 | |
| cpd01468 | EX_HC00900(e) | Methylmalonate | 2.15 | |
| cpd00047 | EX_for(e) | Formate | 26.8 | |
| cpd00106 | EX_fum(e) | Fumarate | 0.7 | |
| cpd00222 | EX_glcn(e) | D-Gluconate | 17.4 | |
| cpd00164 | EX_glcur(e) | Glucuronate | 9.7 | |
| cpd01492 | EX_glutcon(e) | Glutaconate | 3.1 | |
| cpd00379 | EX_glutar(e) | Glutarate | 1.75 | |
| cpd00223 | EX_glyc_R(e) | Glycerate | 15.15 | |
| cpd00100 | EX_glyc(e) | Glycerol | 13 | |
| cpd00139 | EX_glyclt(e) | Glycolate | 40.7 | |
| cpd01114 | EX_bgly(e) | Hippurate | 223 | |
| cpd01606 | EX_hmcr(e) | L-Homocitrulline | 7.5 | |
| cpd00426 | EX_hgentis(e) | Homogentisate | 1.7 | |
| cpd03312 | EX_homoval(e) | Homovanillate | 5.25 | |
| cpd00745 | EX_3hpp(e) | Hydracrylic acid | 10.2 | |

| | | | | |
|---|---|---|---|---|
| cpd00226 | EX_hxan(e) | HYXN | 7.2 | |
| cpd00246 | EX_ins(e) | Inosine | 1.4 | |
| cpd01711 | EX_isobut(e) | Isobutyrate | 3.5 | |
| cpd00260 | EX_icit(e) | Isocitrate | 51.85 | |
| cpd05178 | EX_isoval(e) | Isovalerate | 1.4 | |
| N/A | EX_CE4968(e) | Isovalerylglycine | 2 | |
| cpd01182 | EX_kynate(e) | Kynurenate | 2.35 | |
| cpd00275 | EX_Lkynr(e) | L-Kynurenine | 1.055 | |
| cpd00208 | EX_lcts(e) | LACT | 11.8 | |
| cpd00035 | EX_ala_L(e) | L-Alanine | 21.75 | |
| cpd00224 | EX_arab_L(e) | L-Arabinose | 11.05 | |
| cpd00051 | EX_arg_L(e) | L-Arginine | 8 | |
| cpd00132 | EX_asn_L(e) | L-Asparagine | 9.433333333 | |
| cpd00041 | EX_asp_L(e) | L-Aspartate | 10.9 | |
| cpd00266 | EX_crn(e) | Carnitine | 4.75 | |
| cpd19019 | EX_cyst_L(e) | L-Cystathionine | 14.7 | |
| cpd00084 | EX_cys_L(e) | L-Cysteine | 73.4 | |
| cpd00381 | EX_Lcystin(e) | L-Cystine | 12.5 | |
| cpd00751 | EX_fuc_L(e) | L-Fucose | 11.8 | |
| cpd00023 | EX_glu_L(e) | L-Glutamate | 7.45 | |
| cpd00053 | EX_gln_L(e) | L-Glutamine | 38.55 | |
| cpd00119 | EX_his_L(e) | L-Histidine | 44.3 | |
| cpd00135 | EX_hcys_L(e) | Homocysteine | 1.1 | |
| cpd00322 | EX_ile_L(e) | L-Isoleucine | 1.3 | |
| cpd00159 | EX_lac_L(e) | L-Lactate | 9.35 | |
| cpd00107 | EX_leu_L(e) | L-Leucine | 2.9 | |
| cpd00039 | EX_lys_L(e) | L-Lysine | 17.55 | |
| cpd00060 | EX_met_L(e) | L-Methionine | 1.1 | |
| cpd00066 | EX_phe_L(e) | L-Phenylalanine | 6.7 | |
| cpd00054 | EX_ser_L(e) | L-Serine | 22.93333333 | |
| cpd00161 | EX_thr_L(e) | L-Threonine | 13.95 | |

| | | | | |
|---|---|---|---|---|
| cpd00065 | EX_trp_L(e) | L-Tryptophan | 5.95 | |
| cpd00069 | EX_tyr_L(e) | L-Tyrosine | 11.1 | |
| cpd00156 | EX_val_L(e) | L-Valine | 4.4 | |
| cpd00308 | EX_HC00319(e) | Malonate | 2.6 | |
| cpd00179 | EX_malt(e) | Maltose | 6 | |
| cpd00314 | EX_mnl(e) | D-Mannitol | 32.4 | |
| cpd00116 | EX_meoh(e) | Methanol | 4 | |
| cpd00767 | EX_Nacasp(e) | N-Acetyl-L-aspartate | 4 | |
| cpd00232 | EX_acnam(e) | Neu5Ac | 5.4 | |
| cpd03480 | EX_2hyoxplac(e) | 2-Hydroxyphenylacetate | 2.45 | |
| cpd00024 | EX_akg(e) | 2-Oxoglutarate | 4.1 | |
| cpd00644 | EX_pnto_R(e) | PAN | 1.9 | |
| N/A | EX_pcs(e) | P-Cresol Sulfate | 1.3 | |
| cpd00127 | EX_phenol(e) | Phenol | 4.8 | |
| cpd00430 | EX_pac(e) | PACT | 1.9 | |
| cpd02559 | EX_pheacgln(e) | alpha-N-Phenylacetyl-L-glutamine | 34 | |
| cpd00457 | EX_cholp(e) | Phosphocholine | 1.1 | |
| cpd00489 | EX_4hphac(e) | 4-Hydroxyphenylacetate | 5.5 | |
| cpd07053 | EX_C10164(e) | Picolinic acid | 19.4 | |
| cpd01727 | EX_pime(e) | Pimelate | 2.05 | |
| cpd04117 | EX_4mcat(e) | 4-methylcatechol | 4.7 | |
| cpd00020 | EX_pyr(e) | Pyruvate | 2.4 | |
| cpd02333 | EX_quln(e) | Quinolinate | 4.55 | |
| cpd01187 | EX_sbt_L(e) | D-Gulitol | 6.9 | |
| cpd00036 | EX_succ(e) | Succinate | 6.1 | |
| N/A | EX_sucaceto(e) | Succinylacetone | 2.8 | |
| cpd00076 | EX_sucr(e) | Sucrose | 7.4 | |
| cpd00210 | EX_taur(e) | Taurine | 81 | |

| | | | | |
|---|---|---|---|---|
| cpd01138 | EX_thrnt(e) | Threonate | 17.8 | |
| cpd00184 | EX_thymd(e) | Thymidine | 2.2 | |
| cpd00441 | EX_tma(e) | (CH3)3N | 2.5 | |
| cpd00811 | EX_tmao(e) | (CH3)3NO | 91 | |
| cpd00092 | EX_ura(e) | Uracil | 9.5 | |
| cpd00073 | EX_urea(e) | Urea | 12285 | |
| cpd03346 | EX_5hoxindoa(e) | 5-Hydroxyindoleacetate | 2.9 | |
| N/A | EX_xylu_D(e) | xylu_D | 19.7 | |
| cpd00033 | EX_gly(e) | Glycine | 94.23333333 | |
| cpd02475 | EX_HC02191(e) | Lithocholate | 0.16 | |
| cpd00064 | EX_orn(e) | Ornithine | 4.85 | |
| cpd00009 | EX_pi(e) | Phosphate | 784 | |
| cpd00300 | EX_urate(e) | Urate | 186 | |
| cpd00291 | EX_34dhphe(e) | L-Dopa | 0.02 | |
| cpd00579 | EX_srtn(e) | Serotonin | 0.08 | |
| cpd00274 | EX_citr_L(e) | Citrulline | 0.8 | |
| cpd00129 | EX_pro_L(e) | L-Proline | 1.1 | |
| cpd02357 | EX_dopa(e) | Dopamine | 0.4 | |
| cpd00312 | EX_hista(e) | Histamine | 0.03 | |
| cpd37273 | EX_cd2(e) | Cadmium | 0.0004 | |
| cpd00063 | EX_ca2(e) | Ca2+ | 200 | |
| cpd00149 | EX_cobalt2(e) | Co2+ | 0.0014 | |
| cpd10515 | EX_fe2(e) | Fe+2 | 0.089 | |
| cpd37276 | EX_pb(e) | Lead | 0.0026 | |
| cpd00971 | EX_na1(e) | Na+ | 12477 | |
| cpd00034 | EX_zn2(e) | Zn2+ | 0.46 | |

| prod | cons | met | sim_step | prod.flux | cons.flux | Sample |
|---|---|---|---|---|---|---|
| S_sonnei | L_amylolyticus | EX_cpd00309_e | 3 | 26.11907 | -1.7014 | A01 |
| B_wexlerae | L_amylolyticus | EX_cpd00309_e | 3 | 43.96956 | -1.7014 | A01 |
| P_somerae | L_amylolyticus | EX_cpd00309_e | 3 | 302.3091 | -1.7014 | A01 |
| P_oris | L_amylolyticus | EX_cpd00309_e | 3 | 536.2465 | -1.7014 | A01 |
| B_wexlerae | S_sonnei | EX_cpd00033_ | 3 | 2257.525 | -12.3387 | A01 |
| B_wexlerae | P_uenonsis | EX_cpd00033_e | 3 | 2257.525 | -6.28186 | A01 |
| B_wexlerae | L_johnsonii | EX_cpd00033_e | 3 | 2257.525 | -16.6109 | A01 |
| B_wexlerae | L_crispatus | EX_cpd00033_e | 3 | 2257.525 | -133.296 | A01 |
| B_wexlerae | L_acidophilus | EX_cpd00033_e | 3 | 2257.525 | -29.8487 | A01 |
| B_wexlerae | L_amylolyticus | EX_cpd00033_e | 3 | 2257.525 | -10.6336 | A01 |
| S_sonnei | L_johnsonii | EX_cpd00036_e | 3 | 195.9903 | -0.20729 | A01 |
| P_timonensis | L_johnsonii | EX_cpd00036_e | 3 | 47587.53 | -0.20729 | A01 |
| P_uenonsis | L_johnsonii | EX_cpd00036_e | 3 | 5852.031 | -0.20729 | A01 |
| B_wexlerae | L_johnsonii | EX_cpd00036_e | 3 | 1159.108 | -0.20729 | A01 |
| P_somerae | L_johnsonii | EX_cpd00036_e | 3 | 1622.66 | -0.20729 | A01 |
| P_oris | L_johnsonii | EX_cpd00036_e | 3 | 2878.332 | -0.20729 | A01 |
| L_amylolyticus | L_johnsonii | EX_cpd00036_e | 3 | 0.315246 | -0.20729 | A01 |
| S_sonnei | L_crispatus | EX_cpd00036_e | 3 | 195.9903 | -1.66346 | A01 |
| P_timonensis | L_crispatus | EX_cpd00036_e | 3 | 47587.53 | -1.66346 | A01 |
| P_uenonsis | L_crispatus | EX_cpd00036_e | 3 | 5852.031 | -1.66346 | A01 |
| B_wexlerae | L_crispatus | EX_cpd00036_e | 3 | 1159.108 | -1.66346 | A01 |
| P_somerae | L_crispatus | EX_cpd00036_e | 3 | 1622.66 | -1.66346 | A01 |
| P_oris | L_crispatus | EX_cpd00036_e | 3 | 2878.332 | -1.66346 | A01 |
| L_amylolyticus | L_crispatus | EX_cpd00036_e | 3 | 0.315246 | -1.66346 | A01 |
| S_sonnei | L_acidophilus | EX_cpd00036_e | 3 | 195.9903 | -0.37249 | A01 |
| P_timonensis | L_acidophilus | EX_cpd00036_e | 3 | 47587.53 | -0.37249 | A01 |
| P_uenonsis | L_acidophilus | EX_cpd00036_e | 3 | 5852.031 | -0.37249 | A01 |
| B_wexlerae | L_acidophilus | EX_cpd00036_e | 3 | 1159.108 | -0.37249 | A01 |
| P_somerae | L_acidophilus | EX_cpd00036_e | 3 | 1622.66 | -0.37249 | A01 |
| P_oris | L_acidophilus | EX_cpd00036_e | 3 | 2878.332 | -0.37249 | A01 |
| L_amylolyticus | L_acidophilus | EX_cpd00036_e | 3 | 0.315246 | -0.37249 | A01 |

| | | | | | | |
|---|---|---|---|---|---|---|
| L_amylolyticus | P_uenonsis | EX_cpd00246_e | 3 | 9.713496 | -68.9799 | A01 |
| L_amylolyticus | P_somerae | EX_cpd00246_e | 3 | 9.713496 | -320.223 | A01 |
| L_amylolyticus | P_oris | EX_cpd00246_e | 3 | 9.713496 | -568.023 | A01 |
| L_amylolyticus | B_wexlerae | EX_cpd00020_e | 3 | 5.3927 | -6786 | A01 |
| L_amylolyticus | P_somerae | EX_cpd00020_e | 3 | 5.3927 | -1457.27 | A01 |
| L_amylolyticus | P_oris | EX_cpd00020_e | 3 | 5.3927 | -2584.96 | A01 |
| S_sonnei | L_johnsonii | EX_cpd00036_e | 3 | 154.9269 | -0.20837 | A02 |
| P_timonensis | L_johnsonii | EX_cpd00036_e | 3 | 48793.09 | -0.20837 | A02 |
| P_uenonsis | L_johnsonii | EX_cpd00036_e | 3 | 6105.798 | -0.20837 | A02 |
| B_wexlerae | L_johnsonii | EX_cpd00036_e | 3 | 1136.479 | -0.20837 | A02 |
| UTI89 | L_johnsonii | EX_cpd00036_e | 3 | 779.3134 | -0.20837 | A02 |
| UTI89 | P_timonensis | EX_cpd00082_e | 3 | 1860.788 | -170.173 | A02 |
| B_wexlerae | P_uenonsis | EX_cpd00033_e | 3 | 2213.453 | -6.55427 | A02 |
| B_wexlerae | L_johnsonii | EX_cpd00033_e | 3 | 2213.453 | -16.6971 | A02 |
| S_sonnei | P_lacrimalis | EX_cpd00067_e | 3 | 8205.725 | -28.868 | B01 |
| P_timonensis | P_lacrimalis | EX_cpd00067_e | 3 | 790.4647 | -28.868 | B01 |
| P_dentalis | P_lacrimalis | EX_cpd00067_e | 3 | 564.1165 | -28.868 | B01 |
| P_uenonsis | P_lacrimalis | EX_cpd00067_e | 3 | 147.3722 | -28.868 | B01 |
| L_johnsonii | P_lacrimalis | EX_cpd00067_e | 3 | 86.08719 | -28.868 | B01 |
| UTI89 | P_lacrimalis | EX_cpd00067_e | 3 | 59465.8 | -28.868 | B01 |
| S_sonnei | L_johnsonii | EX_cpd00036_e | 3 | 3019.202 | -0.01394 | B01 |
| P_timonensis | L_johnsonii | EX_cpd00036_e | 3 | 851.9574 | -0.01394 | B01 |
| P_dentalis | L_johnsonii | EX_cpd00036_e | 3 | 342.6071 | -0.01394 | B01 |
| P_uenonsis | L_johnsonii | EX_cpd00036_e | 3 | 82.13526 | -0.01394 | B01 |
| B_wexlerae | L_johnsonii | EX_cpd00036_e | 3 | 7.307211 | -0.01394 | B01 |
| UTI89 | L_johnsonii | EX_cpd00036_e | 3 | 9584.364 | -0.01394 | B01 |
| S_sonnei | P_lacrimalis | EX_cpd00036_e | 3 | 3019.202 | -0.09951 | B01 |
| P_timonensis | P_lacrimalis | EX_cpd00036_e | 3 | 851.9574 | -0.09951 | B01 |
| P_dentalis | P_lacrimalis | EX_cpd00036_e | 3 | 342.6071 | -0.09951 | B01 |
| P_uenonsis | P_lacrimalis | EX_cpd00036_e | 3 | 82.13526 | -0.09951 | B01 |
| B_wexlerae | P_lacrimalis | EX_cpd00036_e | 3 | 7.307211 | -0.09951 | B01 |

| | | | | | | |
|---|---|---|---|---|---|---|
| UTI89 | P_lacrimalis | EX_cpd00036_e | 3 | 9584.364 | -0.09951 | B01 |
| P_timonensis | L_johnsonii | EX_cpd00001_e | 3 | 171.7713 | -3.66188 | B01 |
| P_dentalis | L_johnsonii | EX_cpd00001_e | 3 | 10.25118 | -3.66188 | B01 |
| B_wexlerae | P_uenonsis | EX_cpd00033_e | 3 | 644.1551 | -11.6374 | B02 |
| B_wexlerae | L_johnsonii | EX_cpd00033_e | 3 | 644.1551 | -50.8752 | B02 |
| B_wexlerae | L_crispatus | EX_cpd00033_e | 3 | 644.1551 | -27.3388 | B02 |
| B_wexlerae | L_acidophilus | EX_cpd00033_e | 3 | 644.1551 | -28.2008 | B02 |
| B_wexlerae | L_amylolyticus | EX_cpd00033_e | 3 | 644.1551 | -1.82002 | B02 |
| S_sonnei | L_johnsonii | EX_cpd00036_e | 3 | 39.02119 | -0.63489 | B02 |
| P_timonensis | L_johnsonii | EX_cpd00036_e | 3 | 41301.83 | -0.63489 | B02 |
| P_uenonsis | L_johnsonii | EX_cpd00036_e | 3 | 10841.1 | -0.63489 | B02 |
| B_wexlerae | L_johnsonii | EX_cpd00036_e | 3 | 330.7362 | -0.63489 | B02 |
| UTI89 | L_johnsonii | EX_cpd00036_e | 3 | 37.13448 | -0.63489 | B02 |
| L_amylolyticus | L_johnsonii | EX_cpd00036_e | 3 | 0.053957 | -0.63489 | B02 |
| S_sonnei | L_crispatus | EX_cpd00036_e | 3 | 39.02119 | -0.34117 | B02 |
| P_timonensis | L_crispatus | EX_cpd00036_e | 3 | 41301.83 | -0.34117 | B02 |
| P_uenonsis | L_crispatus | EX_cpd00036_e | 3 | 10841.1 | -0.34117 | B02 |
| B_wexlerae | L_crispatus | EX_cpd00036_e | 3 | 330.7362 | -0.34117 | B02 |
| UTI89 | L_crispatus | EX_cpd00036_e | 3 | 37.13448 | -0.34117 | B02 |
| L_amylolyticus | L_crispatus | EX_cpd00036_e | 3 | 0.053957 | -0.34117 | B02 |
| S_sonnei | L_acidophilus | EX_cpd00036_e | 3 | 39.02119 | -0.35193 | B02 |
| P_timonensis | L_acidophilus | EX_cpd00036_e | 3 | 41301.83 | -0.35193 | B02 |
| P_uenonsis | L_acidophilus | EX_cpd00036_e | 3 | 10841.1 | -0.35193 | B02 |
| B_wexlerae | L_acidophilus | EX_cpd00036_e | 3 | 330.7362 | -0.35193 | B02 |
| UTI89 | L_acidophilus | EX_cpd00036_e | 3 | 37.13448 | -0.35193 | B02 |
| L_amylolyticus | L_acidophilus | EX_cpd00036_e | 3 | 0.053957 | -0.35193 | B02 |
| P_timonensis | L_johnsonii | EX_cpd00001_e | 3 | 8327.259 | -146.094 | B02 |
| P_timonensis | L_crispatus | EX_cpd00001_e | 3 | 8327.259 | -78.5067 | B02 |
| P_timonensis | L_acidophilus | EX_cpd00001_e | 3 | 8327.259 | -80.9821 | B02 |
| P_timonensis | L_amylolyticus | EX_cpd00001_e | 3 | 8327.259 | -3.55806 | B02 |
| S_sonnei | P_uenonsis | EX_cpd00041_e | 3 | 35.23928 | -365.948 | C01 |

| | | | | | | |
|---|---|---|---|---|---|---|
| S_sonnei | B_wexlerae | EX_cpd00041_e | 3 | 35.23928 | -9.30295 | C01 |
| S_sonnei | L_johnsonii | EX_cpd00041_e | 3 | 35.23928 | -1.48752 | C01 |
| S_sonnei | P_oris | EX_cpd00041_e | 3 | 35.23928 | -2328.87 | C01 |
| S_sonnei | L_johnsonii | EX_cpd00036_e | 3 | 690.382 | -0.07101 | C01 |
| P_timonensis | L_johnsonii | EX_cpd00036_e | 3 | 7701.533 | -0.07101 | C01 |
| P_uenonsis | L_johnsonii | EX_cpd00036_e | 3 | 13258.5 | -0.07101 | C01 |
| B_wexlerae | L_johnsonii | EX_cpd00036_e | 3 | 487.6981 | -0.07101 | C01 |
| P_oris | L_johnsonii | EX_cpd00036_e | 3 | 11312.27 | -0.07101 | C01 |
| UTI89 | L_johnsonii | EX_cpd00036_e | 3 | 10063.81 | -0.07101 | C01 |
| P_timonensis | L_johnsonii | EX_cpd00001_e | 3 | 1552.78 | -17.6632 | C01 |
| P_uenonsis | L_johnsonii | EX_cpd00001_e | 3 | 373.2486 | -17.6632 | C01 |
| P_uenonsis | S_sonnei | EX_cpd00211_e | 3 | 0.021567 | -86.3195 | C02 |
| UTI89 | S_sonnei | EX_cpd00211_e | 3 | 16956.92 | -86.3195 | C02 |
| S_sonnei | L_johnsonii | EX_cpd00036_e | 3 | 20.56531 | -0.01242 | D01 |
| P_timonensis | L_johnsonii | EX_cpd00036_e | 3 | 50763.38 | -0.01242 | D01 |
| P_uenonsis | L_johnsonii | EX_cpd00036_e | 3 | 8894.774 | -0.01242 | D01 |
| B_wexlerae | L_johnsonii | EX_cpd00036_e | 3 | 1050.277 | -0.01242 | D01 |
| L_crispatus | L_johnsonii | EX_cpd00036_e | 3 | 0.280335 | -0.01242 | D01 |
| UTI89 | L_johnsonii | EX_cpd00036_e | 3 | 187.1444 | -0.01242 | D01 |
| L_acidophilus | L_johnsonii | EX_cpd00036_e | 3 | 4.931608 | -0.01242 | D01 |
| P_uenonsis | S_sonnei | EX_cpd00211_e | 3 | 5.123232 | -1.01355 | D01 |
| UTI89 | S_sonnei | EX_cpd00211_e | 3 | 563.2639 | -1.01355 | D01 |
| B_wexlerae | L_johnsonii | EX_cpd00033_e | 3 | 2045.562 | -0.99551 | D01 |
| B_wexlerae | L_crispatus | EX_cpd00033_e | 3 | 2045.562 | -9.45603 | D01 |
| B_wexlerae | L_amylolyticus | EX_cpd00033_e | 3 | 2045.562 | -2.20238 | D01 |
| L_acidophilus | S_sonnei | EX_cpd00161_e | 3 | 2.417349 | -598.799 | D02 |
| L_acidophilus | P_timonensis | EX_cpd00161_e | 3 | 2.417349 | -1.82979 | D02 |
| L_acidophilus | P_uenonsis | EX_cpd00161_e | 3 | 2.417349 | -1.0143 | D02 |
| L_acidophilus | B_wexlerae | EX_cpd00161_e | 3 | 2.417349 | -1.01005 | D02 |
| L_acidophilus | D_invisus | EX_cpd00161_e | 3 | 2.417349 | -0.78612 | D02 |
| L_acidophilus | L_iners | EX_cpd00161_e | 3 | 2.417349 | -0.42223 | D02 |

| | | | | | | |
|---|---|---|---|---|---|---|
| L_acidophilus | L_johnsonii | EX_cpd00161_e | 3 | 2.417349 | -1.33844 | D02 |
| L_acidophilus | L_crispatus | EX_cpd00161_e | 3 | 2.417349 | -0.37873 | D02 |
| L_acidophilus | P_lacrimalis | EX_cpd00161_e | 3 | 2.417349 | -0.38615 | D02 |
| L_acidophilus | P_oris | EX_cpd00161_e | 3 | 2.417349 | -75.819 | D02 |
| L_acidophilus | UTI89 | EX_cpd00161_e | 3 | 2.417349 | -393.896 | D02 |
| L_acidophilus | L_amylolyticus | EX_cpd00161_e | 3 | 2.417349 | -0.97084 | D02 |
| B_wexlerae | P_oris | EX_cpd00309_e | 3 | 0.27021 | -37.9819 | D02 |
| UTI89 | P_oris | EX_cpd00309_e | 3 | 2389.373 | -37.9819 | D02 |
| S_sonnei | D_invisus | EX_cpd00036_e | 3 | 2494.065 | -0.02412 | D02 |
| P_timonensis | D_invisus | EX_cpd00036_e | 3 | 368.4608 | -0.02412 | D02 |
| P_uenonsis | D_invisus | EX_cpd00036_e | 3 | 86.58166 | -0.02412 | D02 |
| B_wexlerae | D_invisus | EX_cpd00036_e | 3 | 7.123177 | -0.02412 | D02 |
| L_iners | D_invisus | EX_cpd00036_e | 3 | 0.030177 | -0.02412 | D02 |
| L_johnsonii | D_invisus | EX_cpd00036_e | 3 | 4.326995 | -0.02412 | D02 |
| L_crispatus | D_invisus | EX_cpd00036_e | 3 | 5.80E-01 | -0.02412 | D02 |
| P_oris | D_invisus | EX_cpd00036_e | 3 | 5.42E+00 | -0.02412 | D02 |
| UTI89 | D_invisus | EX_cpd00036_e | 3 | 1.30E+04 | -0.02412 | D02 |
| B_wexlerae | D_invisus | EX_cpd00033_e | 3 | 1.39E+01 | -1.93265 | D02 |
| B_wexlerae | L_iners | EX_cpd00033_e | 3 | 13.87339 | -1.01792 | D02 |
| B_wexlerae | P_lacrimalis | EX_cpd00033_e | 3 | 13.87339 | -0.95857 | D02 |
| B_wexlerae | P_oris | EX_cpd00033_e | 3 | 13.87339 | -182.787 | D02 |
| B_wexlerae | UTI89 | EX_cpd00033_e | 3 | 13.87339 | -888.692 | D02 |
| B_wexlerae | L_amylolyticus | EX_cpd00033_e | 3 | 13.87339 | -2.3989 | D02 |
| UTI89 | S_sonnei | EX_cpd00051_e | 3 | 364672.9 | -23140.6 | E01 |
| UTI89 | P_timonensis | EX_cpd00051_e | 3 | 364672.9 | -3.76834 | E01 |
| UTI89 | P_uenonsis | EX_cpd00051_e | 3 | 364672.9 | -3.79627 | E01 |
| UTI89 | B_wexlerae | EX_cpd00051_e | 3 | 364672.9 | -6.1583 | E01 |
| UTI89 | S_sonnei | EX_cpd00107_e | 3 | 1173179 | -16715.6 | E01 |
| UTI89 | P_timonensis | EX_cpd00107_e | 3 | 1173179 | -6.66928 | E01 |
| UTI89 | P_uenonsis | EX_cpd00107_e | 3 | 1173179 | -6.90758 | E01 |
| UTI89 | B_wexlerae | EX_cpd00107_e | 3 | 1173179 | -8.16283 | E01 |

| | | | | | | |
|---|---|---|---|---|---|---|
| S_sonnei | P_timonensis | EX_cpd00092_e | 3 | 2985.498 | -487.509 | E01 |
| UTI89 | P_timonensis | EX_cpd00092_e | 3 | 83043.81 | -487.509 | E01 |
| S_sonnei | B_wexlerae | EX_cpd00092_e | 3 | 2985.498 | -2.10288 | E01 |
| UTI89 | B_wexlerae | EX_cpd00092_e | 3 | 83043.81 | -2.10288 | E01 |
| UTI89 | S_sonnei | EX_cpd00069_e | 3 | 100356 | -22.3391 | E01 |
| UTI89 | P_uenonsis | EX_cpd00069_e | 3 | 100356 | -596.05 | E01 |
| UTI89 | B_wexlerae | EX_cpd00069_e | 3 | 100356 | -2.24242 | E01 |
| UTI89 | S_sonnei | EX_cpd01217_e | 3 | 1767176 | -21.0036 | E01 |
| UTI89 | P_timonensis | EX_cpd01217_e | 3 | 1767176 | -993.731 | E01 |
| P_timonensis | B_wexlerae | EX_cpd00023_e | 3 | 13000 | -20.7368 | E01 |
| UTI89 | B_wexlerae | EX_cpd00023_e | 3 | 28695.81 | -20.7368 | E01 |
| S_sonnei | P_lacrimalis | EX_cpd00067_e | 3 | 53377.94 | -69.325 | E02 |
| P_timonensis | P_lacrimalis | EX_cpd00067_e | 3 | 77.26406 | -69.325 | E02 |
| UTI89 | P_lacrimalis | EX_cpd00067_e | 3 | 25904.33 | -69.325 | E02 |
| S_sonnei | P_lacrimalis | EX_cpd00036_e | 3 | 11806.33 | -0.23897 | E02 |
| P_timonensis | P_lacrimalis | EX_cpd00036_e | 3 | 83.27467 | -0.23897 | E02 |
| UTI89 | P_lacrimalis | EX_cpd00036_e | 3 | 3783.823 | -0.23897 | E02 |
| S_sonnei | UTI89 | EX_cpd00180_e | 3 | 6651.312 | -3.41498 | E02 |
| UTI89 | P_lacrimalis | EX_cpd00020_e | 3 | 16419.5 | -208.407 | E02 |
| UTI89 | P_dentalis | EX_cpd00179_e | 3 | 260252.3 | -3636.36 | F01 |
| UTI89 | P_oris | EX_cpd00179_e | 3 | 260252.3 | -82668.5 | F01 |
| S_sonnei | UTI89 | EX_cpd00309_e | 3 | 124496.6 | -59759.3 | F01 |
| P_timonensis | UTI89 | EX_cpd00309_e | 3 | 3907.731 | -59759.3 | F01 |
| B_wexlerae | UTI89 | EX_cpd00309_e | 3 | 2.236559 | -59759.3 | F01 |
| S_sonnei | P_timonensis | EX_cpd00054_e | 3 | 163654.2 | -570.419 | F01 |
| S_sonnei | B_wexlerae | EX_cpd00054_e | 3 | 163654.2 | -330.62 | F01 |
| S_sonnei | P_timonensis | EX_cpd00644_e | 3 | 127510.5 | -0.10672 | F01 |
| UTI89 | P_timonensis | EX_cpd00644_e | 3 | 223350.7 | -0.10672 | F01 |
| S_sonnei | P_dentalis | EX_cpd00644_e | 3 | 127510.5 | -0.14357 | F01 |
| UTI89 | P_dentalis | EX_cpd00644_e | 3 | 223350.7 | -0.14357 | F01 |
| S_sonnei | B_wexlerae | EX_cpd00644_e | 3 | 127510.5 | -0.14277 | F01 |

| | | | | | | |
|---|---|---|---|---|---|---|
| UTI89 | B_wexlerae | EX_cpd00644_e | 3 | 223350.7 | -0.14277 | F01 |
| S_sonnei | P_oris | EX_cpd00644_e | 3 | 127510.5 | -3.43105 | F01 |
| UTI89 | P_oris | EX_cpd00644_e | 3 | 223350.7 | -3.43105 | F01 |
| S_sonnei | P_dentalis | EX_cpd00029_e | 3 | 462000 | -7275.41 | F01 |
| P_timonensis | P_dentalis | EX_cpd00029_e | 3 | 14000 | -7275.41 | F01 |
| B_wexlerae | P_dentalis | EX_cpd00029_e | 3 | 632.4281 | -7275.41 | F01 |
| P_oris | P_dentalis | EX_cpd00029_e | 3 | 436000 | -7275.41 | F01 |
| UTI89 | P_dentalis | EX_cpd00029_e | 3 | 1981000 | -7275.41 | F01 |
| P_oris | S_sonnei | EX_cpd00080_e | 3 | 131006.2 | -100.404 | F01 |
| B_wexlerae | S_sonnei | EX_cpd00033_e | 3 | 320.1481 | -462000 | F01 |
| B_wexlerae | P_dentalis | EX_cpd00033_e | 3 | 320.1481 | -17.7856 | F01 |
| S_sonnei | P_oris | EX_cpd00036_e | 3 | 381280.5 | -808.704 | F01 |
| P_timonensis | P_oris | EX_cpd00036_e | 3 | 14000 | -808.704 | F01 |
| P_dentalis | P_oris | EX_cpd00036_e | 3 | 5803.195 | -808.704 | F01 |
| B_wexlerae | P_oris | EX_cpd00036_e | 3 | 58.95928 | -808.704 | F01 |
| UTI89 | P_oris | EX_cpd00036_e | 3 | 1713507 | -808.704 | F01 |
| P_oris | S_sonnei | EX_cpd00221_e | 3 | 248073.8 | -9293.11 | F01 |
| P_oris | P_timonensis | EX_cpd00221_e | 3 | 248073.8 | -281.609 | F01 |
| UTI89 | P_timonensis | EX_cpd00092_e | 3 | 1757064 | -4.2308 | F01 |
| UTI89 | P_dentalis | EX_cpd00092_e | 3 | 1757064 | -0.14357 | F01 |
| UTI89 | B_wexlerae | EX_cpd00092_e | 3 | 1757064 | -1.73686 | F01 |
| P_timonensis | B_wexlerae | EX_cpd00047_e | 3 | 216.1333 | -0.52024 | F01 |
| P_oris | B_wexlerae | EX_cpd00047_e | 3 | 302465.1 | -0.52024 | F01 |
| P_dentalis | P_timonensis | EX_cpd00106_e | 3 | 13359.06 | -73.3962 | F01 |
| P_dentalis | P_oris | EX_cpd00106_e | 3 | 13359.06 | -184023 | F01 |
| UTI89 | P_timonensis | EX_cpd01217_e | 3 | 554135 | -350.767 | F01 |
| UTI89 | P_timonensis | EX_cpd00129_e | 3 | 868055 | -2.83718 | F01 |
| UTI89 | P_dentalis | EX_cpd00129_e | 3 | 868055 | -3.81676 | F01 |
| UTI89 | B_wexlerae | EX_cpd00129_e | 3 | 868055 | -2.58239 | F01 |
| UTI89 | P_oris | EX_cpd00129_e | 3 | 868055 | -91.2119 | F01 |
| UTI89 | S_sonnei | EX_cpd00051_e | 3 | 364672.9 | -23140.6 | F02 |

| | | | | | | |
|---|---|---|---|---|---|---|
| UTI89 | P_timonensis | EX_cpd00051_e | 3 | 364672.9 | -3.76834 | F02 |
| UTI89 | P_uenonsis | EX_cpd00051_e | 3 | 364672.9 | -3.79627 | F02 |
| UTI89 | B_wexlerae | EX_cpd00051_e | 3 | 364672.9 | -6.1583 | F02 |
| UTI89 | S_sonnei | EX_cpd00107_e | 3 | 1173179 | -16715.6 | F02 |
| UTI89 | P_timonensis | EX_cpd00107_e | 3 | 1173179 | -6.66928 | F02 |
| UTI89 | P_uenonsis | EX_cpd00107_e | 3 | 1173179 | -6.90758 | F02 |
| UTI89 | B_wexlerae | EX_cpd00107_e | 3 | 1173179 | -8.16283 | F02 |
| S_sonnei | P_timonensis | EX_cpd00092_e | 3 | 2985.498 | -487.509 | F02 |
| UTI89 | P_timonensis | EX_cpd00092_e | 3 | 83043.81 | -487.509 | F02 |
| S_sonnei | B_wexlerae | EX_cpd00092_e | 3 | 2985.498 | -2.10288 | F02 |
| UTI89 | B_wexlerae | EX_cpd00092_e | 3 | 83043.81 | -2.10288 | F02 |
| UTI89 | S_sonnei | EX_cpd00069_e | 3 | 100356 | -22.3391 | F02 |
| UTI89 | P_uenonsis | EX_cpd00069_e | 3 | 100356 | -596.05 | F02 |
| UTI89 | B_wexlerae | EX_cpd00069_e | 3 | 100356 | -2.24242 | F02 |
| UTI89 | S_sonnei | EX_cpd01217_e | 3 | 1767176 | -21.0036 | F02 |
| UTI89 | P_timonensis | EX_cpd01217_e | 3 | 1767176 | -993.731 | F02 |
| P_timonensis | B_wexlerae | EX_cpd00023_e | 3 | 13000 | -20.7368 | F02 |
| UTI89 | B_wexlerae | EX_cpd00023_e | 3 | 28695.81 | -20.7368 | F02 |
| L_acidophilus | S_sonnei | EX_cpd00161_e | 3 | 35.65227 | -4.87399 | G01 |
| L_acidophilus | P_timonensis | EX_cpd00161_e | 3 | 35.65227 | -21.4701 | G01 |
| L_acidophilus | P_uenonsis | EX_cpd00161_e | 3 | 35.65227 | -30.6678 | G01 |
| L_acidophilus | B_wexlerae | EX_cpd00161_e | 3 | 35.65227 | -17.5063 | G01 |
| L_acidophilus | B_intestinihomir | EX_cpd00161_e | 3 | 35.65227 | -82.1326 | G01 |
| L_acidophilus | D_invisus | EX_cpd00161_e | 3 | 35.65227 | -224.779 | G01 |
| L_acidophilus | A_geminatus | EX_cpd00161_e | 3 | 35.65227 | -195.579 | G01 |
| L_acidophilus | L_johnsonii | EX_cpd00161_e | 3 | 35.65227 | -25.1895 | G01 |
| L_acidophilus | P_lacrimalis | EX_cpd00161_e | 3 | 35.65227 | -33.7538 | G01 |
| L_acidophilus | P_oris | EX_cpd00161_e | 3 | 35.65227 | -0.91706 | G01 |
| L_acidophilus | UTI89 | EX_cpd00161_e | 3 | 35.65227 | -11.0144 | G01 |
| S_sonnei | B_intestinihomir | EX_cpd00036_e | 3 | 20.30075 | -2.51985 | G01 |
| P_timonensis | B_intestinihomir | EX_cpd00036_e | 3 | 4323.393 | -2.51985 | G01 |

| | | | | | | |
|---|---|---|---|---|---|---|
| P_uenonsis | B_intestinihomir | EX_cpd00036_e | 3 | 2617.845 | -2.51985 | G01 |
| B_wexlerae | B_intestinihomir | EX_cpd00036_e | 3 | 123.4598 | -2.51985 | G01 |
| D_invisus | B_intestinihomir | EX_cpd00036_e | 3 | 16.06541 | -2.51985 | G01 |
| A_geminatus | B_intestinihomir | EX_cpd00036_e | 3 | 632.278 | -2.51985 | G01 |
| L_johnsonii | B_intestinihomir | EX_cpd00036_e | 3 | 3.86E+01 | -2.51985 | G01 |
| P_oris | B_intestinihomir | EX_cpd00036_e | 3 | 6.55E-02 | -2.51985 | G01 |
| UTI89 | B_intestinihomir | EX_cpd00036_e | 3 | 3.63E+02 | -2.51985 | G01 |
| P_uenonsis | S_sonnei | EX_cpd00211_e | 3 | 1.51E+00 | -1.00051 | G01 |
| A_geminatus | S_sonnei | EX_cpd00211_e | 3 | 1903.019 | -1.00051 | G01 |
| B_wexlerae | B_intestinihomir | EX_cpd00033_e | 3 | 240.4552 | -201.921 | G01 |
| B_wexlerae | D_invisus | EX_cpd00033_e | 3 | 240.4552 | -541.904 | G01 |
| B_wexlerae | P_lacrimalis | EX_cpd00033_e | 3 | 240.4552 | -83.7903 | G01 |
| B_wexlerae | P_oris | EX_cpd00033_e | 3 | 240.4552 | -2.21088 | G01 |
| B_wexlerae | UTI89 | EX_cpd00033_e | 3 | 240.4552 | -24.8503 | G01 |
| P_oris | B_wexlerae | EX_cpd00020_e | 3 | 1.121219 | -722.795 | G01 |
| UTI89 | S_sonnei | EX_cpd00211_e | 3 | 3418.681 | -19.6342 | H01 |
| B_wexlerae | P_dentalis | EX_cpd00033_e | 3 | 236.5805 | -127.421 | H01 |
| B_wexlerae | UTI89 | EX_cpd00033_e | 3 | 236.5805 | -764.697 | H01 |
| S_sonnei | L_johnsonii | EX_cpd00036_e | 3 | 1509.173 | -0.01199 | H25361 |
| P_timonensis | L_johnsonii | EX_cpd00036_e | 3 | 4127.401 | -0.01199 | H25361 |
| B_wexlerae | L_johnsonii | EX_cpd00036_e | 3 | 50.15222 | -0.01199 | H25361 |
| UTI89 | L_johnsonii | EX_cpd00036_e | 3 | 14833.67 | -0.01199 | H25361 |
| S_sonnei | UTI89 | EX_cpd00053_e | 3 | 92.25766 | -2247.78 | H25362 |
| S_sonnei | P_timonensis | EX_cpd00132_e | 3 | 199.7898 | -12.9328 | H25362 |
| S_sonnei | UTI89 | EX_cpd00132_e | 3 | 199.7898 | -551.131 | H25362 |
| P_timonensis | S_sonnei | EX_cpd00309_e | 3 | 8923.598 | -679.405 | H25363 |
| B_wexlerae | S_sonnei | EX_cpd00309_e | 3 | 8.102467 | -679.405 | H25363 |
| UTI89 | S_sonnei | EX_cpd00309_e | 3 | 266741.2 | -679.405 | H25363 |
| P_timonensis | S_sonnei | EX_cpd00130_e | 3 | 1771000 | -2266.4 | H25363 |
| UTI89 | S_sonnei | EX_cpd00130_e | 3 | 350804.7 | -2266.4 | H25363 |
| S_sonnei | P_timonensis | EX_cpd00054_e | 3 | 9319.733 | -88767.2 | H25363 |

| | | | | | | |
|---|---|---|---|---|---|---|
| S_sonnei | B_wexlerae | EX_cpd00054_e | 3 | 9319.733 | -1197.75 | H25363 |
| S_sonnei | UTI89 | EX_cpd00054_e | 3 | 9319.733 | -133448 | H25363 |
| UTI89 | S_sonnei | EX_cpd00644_e | 3 | 137716.9 | -1754.68 | H25363 |
| UTI89 | P_timonensis | EX_cpd00644_e | 3 | 137716.9 | -14.0243 | H25363 |
| UTI89 | B_wexlerae | EX_cpd00644_e | 3 | 137716.9 | -0.51722 | H25363 |
| UTI89 | P_oris | EX_cpd00644_e | 3 | 137716.9 | -0.14803 | H25363 |
| P_timonensis | S_sonnei | EX_cpd00182_e | 3 | 21.03645 | -2180.83 | H25363 |
| P_timonensis | B_wexlerae | EX_cpd00182_e | 3 | 21.03645 | -19.7442 | H25363 |
| P_timonensis | P_oris | EX_cpd00182_e | 3 | 21.03645 | -4901.02 | H25363 |
| P_timonensis | UTI89 | EX_cpd00182_e | 3 | 21.03645 | -266960 | H25363 |
| S_sonnei | UTI89 | EX_cpd00036_e | 3 | 2655.869 | -493044 | H25363 |
| P_timonensis | UTI89 | EX_cpd00036_e | 3 | 1770993 | -493044 | H25363 |
| B_wexlerae | UTI89 | EX_cpd00036_e | 3 | 213.594 | -493044 | H25363 |
| P_oris | UTI89 | EX_cpd00036_e | 3 | 2.02E+03 | -493044 | H25363 |
| P_oris | P_timonensis | EX_cpd00246_e | 3 | 3.27E+03 | -61259.9 | H25363 |
| P_oris | B_wexlerae | EX_cpd00047_e | 3 | 12939.72 | -1.88469 | H25363 |
| P_timonensis | B_wexlerae | EX_cpd00023_e | 3 | 1753523 | -75.3958 | H25363 |
| P_timonensis | P_oris | EX_cpd00023_e | 3 | 1753523 | -9.92455 | H25363 |
| S_sonnei | UTI89 | EX_cpd00064_e | 3 | 49451.3 | -15.9749 | H25364 |
| UTI89 | S_sonnei | EX_cpd00119_e | 3 | 227985.2 | -25.2207 | H25364 |
| UTI89 | P_timonensis | EX_cpd00119_e | 3 | 227985.2 | -10060.1 | H25364 |
| S_sonnei | P_timonensis | EX_cpd00156_e | 3 | 201444.2 | -21.8345 | H25364 |
| UTI89 | S_sonnei | EX_cpd00161_e | 3 | 549176.6 | -231669 | H25364 |
| UTI89 | P_timonensis | EX_cpd00161_e | 3 | 549176.6 | -16845.4 | H25364 |
| S_sonnei | UTI89 | EX_cpd00179_e | 3 | 140467.1 | -1951607 | H25364 |
| S_sonnei | P_timonensis | EX_cpd00092_e | 3 | 3014.405 | -15.667 | H25364 |
| UTI89 | P_timonensis | EX_cpd00092_e | 3 | 1950160 | -15.667 | H25364 |
| S_sonnei | UTI89 | EX_cpd00054_e | 3 | 281000 | -2361000 | H25364 |
| S_sonnei | P_timonensis | EX_cpd00232_e | 3 | 14590.93 | -402.112 | H25364 |
| S_sonnei | UTI89 | EX_cpd00232_e | 3 | 14590.93 | -317534 | H25364 |
| S_sonnei | UTI89 | EX_cpd00314_e | 3 | 281000 | -284702 | H25364 |

| | | | | | | | |
|---|---|---|---|---|---|---|---|
| UTI89 | S_sonnei | EX_cpd00550_e | 3 | 2360207 | -53369.2 | H25364 | |
| UTI89 | S_sonnei | EX_cpd00176_e | 3 | 2361000 | -44428.2 | H25364 | |
| UTI89 | S_sonnei | EX_cpd00039_e | 3 | 247806 | -12306.4 | H25365 | |
| UTI89 | S_sonnei | EX_cpd00322_e | 3 | 50238.08 | -72.621 | H25365 | |
| UTI89 | P_timonensis | EX_cpd00322_e | 3 | 50238.08 | -26.4439 | H25365 | |
| UTI89 | S_sonnei | EX_cpd00084_e | 3 | 1230499 | -203000 | H25365 | |
| UTI89 | P_timonensis | EX_cpd00084_e | 3 | 1230499 | -37407 | H25365 | |
| UTI89 | S_sonnei | EX_cpd00132_e | 3 | 11787.48 | -46.2851 | H25365 | |
| S_sonnei | UTI89 | EX_cpd00307_e | 3 | 13531.86 | -91991.6 | H25365 | |
| S_sonnei | UTI89 | EX_cpd00314_e | 3 | 13462.87 | -380860 | H25365 | |
| UTI89 | P_timonensis | EX_cpd01217_e | 3 | 492931.5 | -5435.66 | H25365 | |
