# Supplementary material for: Metatranscriptomics-based metabolic modeling of patient-specific urinary microbiome during infection
Source: NPJ Biofilms Microbiomes. 2025 Sep 9;11:183. doi: 10.1038/s41522-025-00823-6 (PMC12420794; doi:10.1038/s41522-025-00823-6)
Supplement: Supplementary file 2 — Uromicrobiome_Supp_Figures. [file 41522_2025_823_MOESM2_ESM.pdf]

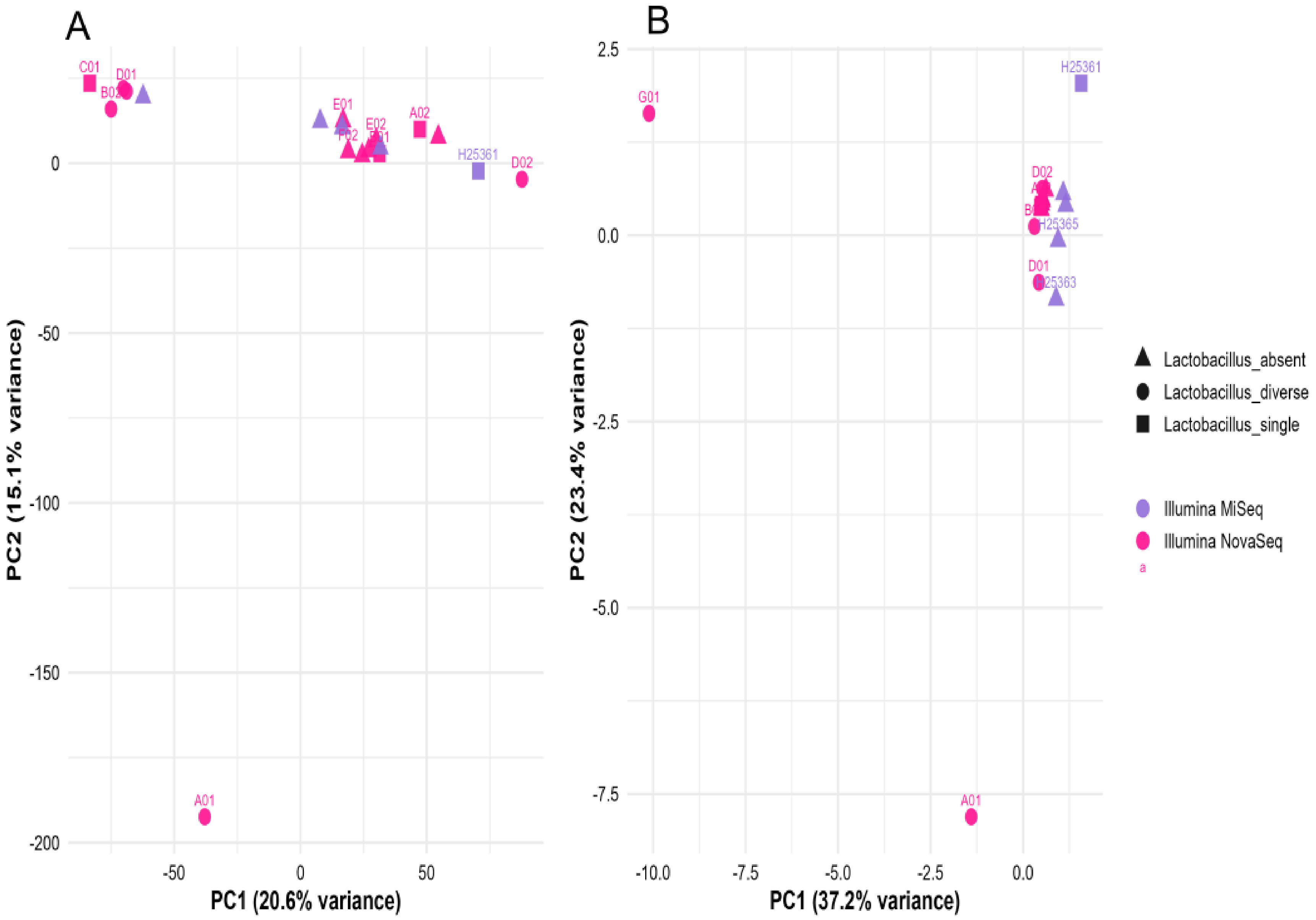
A
B
C01
D01
B02
E01
F02
E02
A02
H25361
D02
A01
G01
H25365
H25363
PC1 (20.6% variance)
PC2 (15.1% variance)
PC1 (37.2% variance)
PC2 (23.4% variance)
Lactobacillus_absent
Lactobacillus_diverse
Lactobacillus_single
Illumina MiSeq
Illumina NovaSeq
a

Supplementary Figure S1: **PCA of OTUs and TUs by Sequencing Technology**

(A) PCA of post-filtered OTUs illustrates microbial community variation, colored by sequencing technology: Illumina MiSeq (purple circles) and NovaSeq (red triangles). (B) PCA of gene expression mapped to identified taxa, shows transcriptional variance across samples, also grouped by sequencing technology

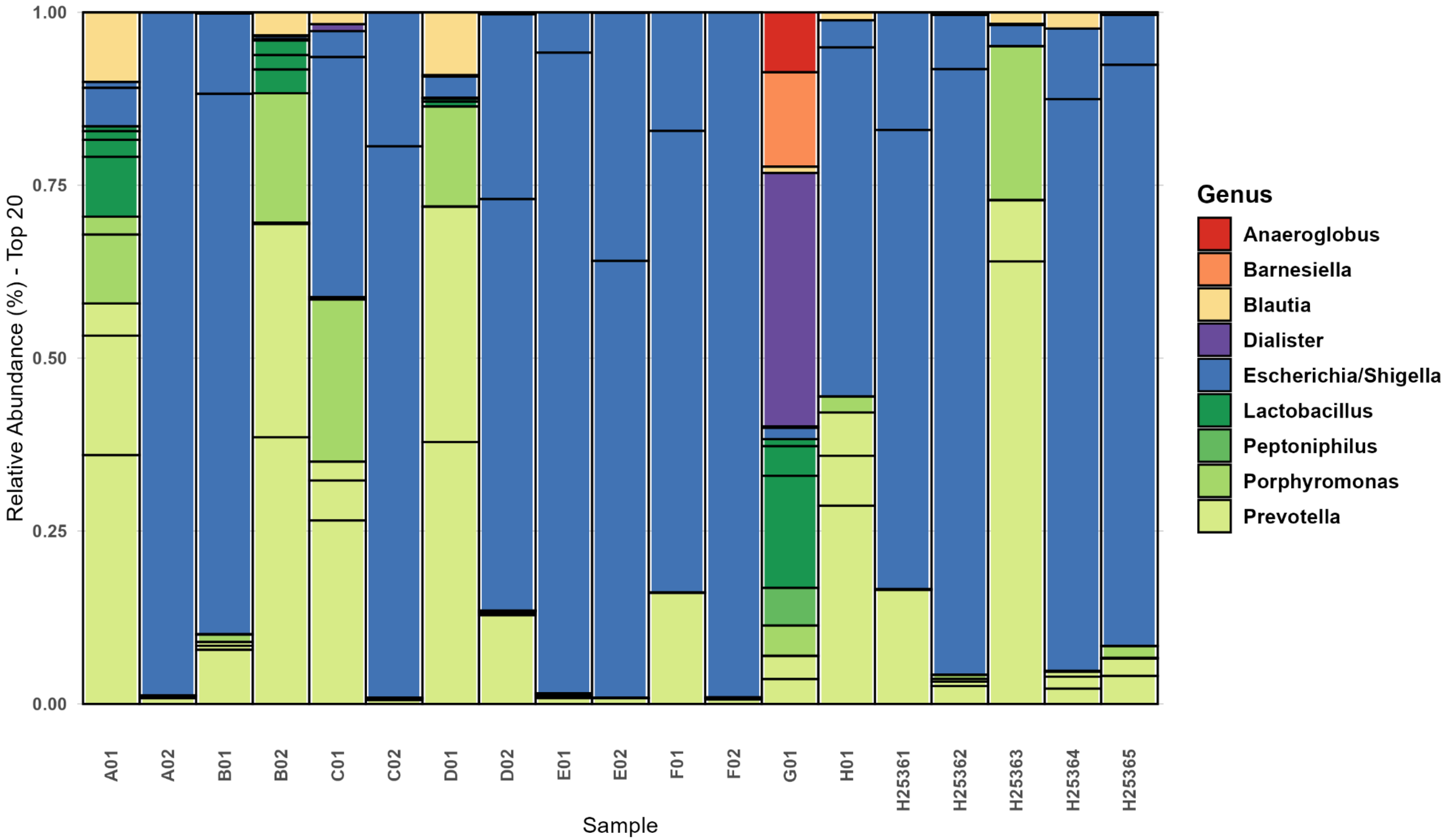
Relative Abundance (%) - Top 20
1.00
0.75
0.50
0.25
0.00
A01
A02
B01
B02
C01
C02
D01
D02
E01
E02
F01
F02
G01
H01
H25361
H25362
H25363
H25364
H25365
Sample
Genus
Anaeroglobus
Barnesiella
Blautia
Dialister
Escherichia/Shigella
Lactobacillus
Peptoniphilus
Porphyromonas
Prevotella

Supplementary Figure S2: **Taxonomic Abundance of Key UTI-Associated Genera**

(A) Abundance profiles of nine pathogenic genera across patient samples, highlighting the variability and patient-specific microbial signatures of UTI microbiomes.

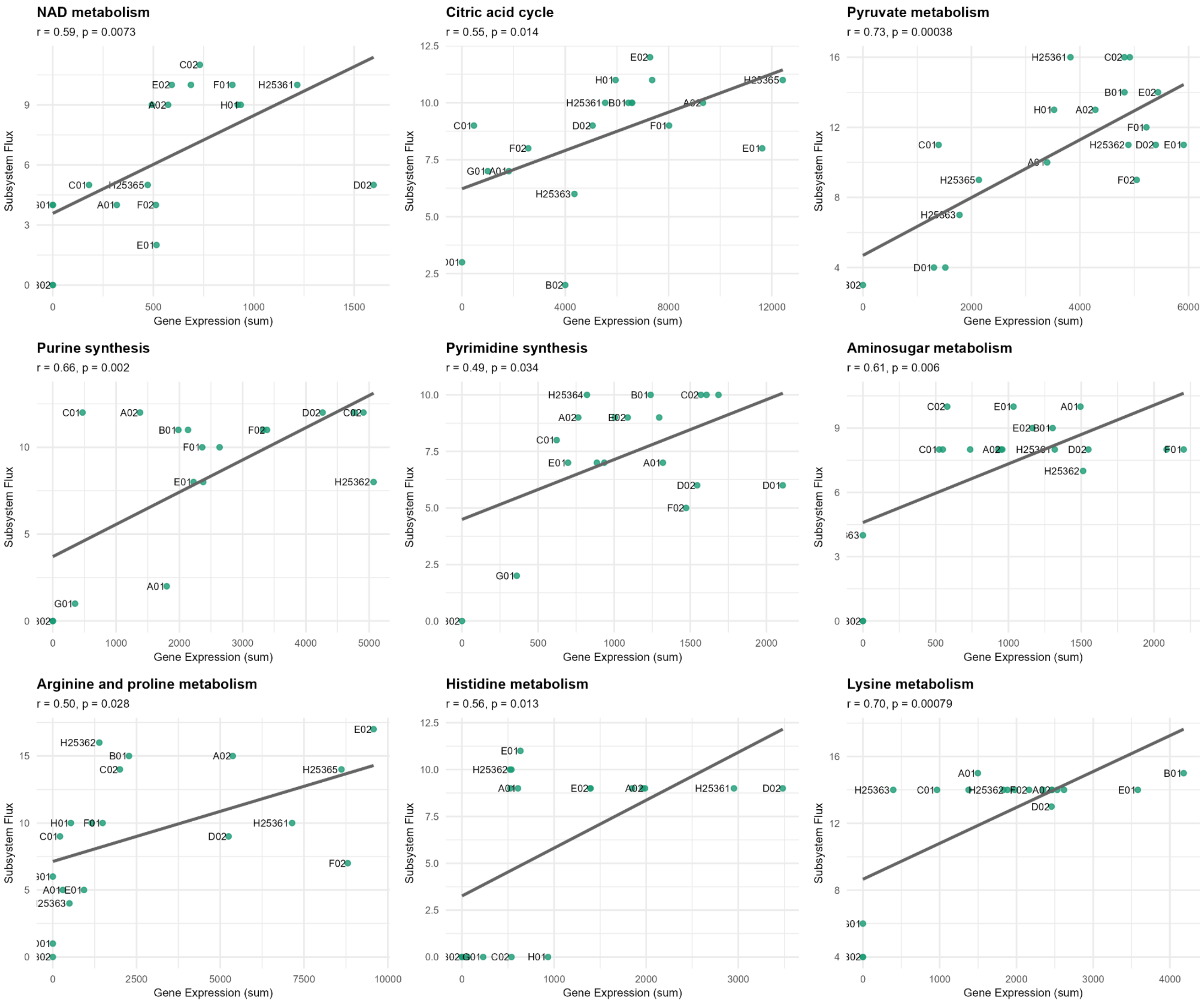
NAD metabolism
r = 0.59, p = 0.0073
Citric acid cycle
r = 0.55, p = 0.014
Pyruvate metabolism
r = 0.73, p = 0.00038
Purine synthesis
r = 0.66, p = 0.002
Pyrimidine synthesis
r = 0.49, p = 0.034
Aminosugar metabolism
r = 0.61, p = 0.006
Arginine and proline metabolism
r = 0.50, p = 0.028
Histidine metabolism
r = 0.56, p = 0.013
Lysine metabolism
r = 0.70, p = 0.00079
Subsystem Flux
Gene Expression (sum)

Supplementary Figure S3. Correlation of summed transcript abundance and predicted flux for nine key metabolic subsystems in *E. coli* UTI89. For each subsystem, patient-specific transcript counts (mapped to both the UTI89 genome and its context-specific metabolic model) are summed and plotted against the model's predicted flux, with each point labeled by its sample ID.

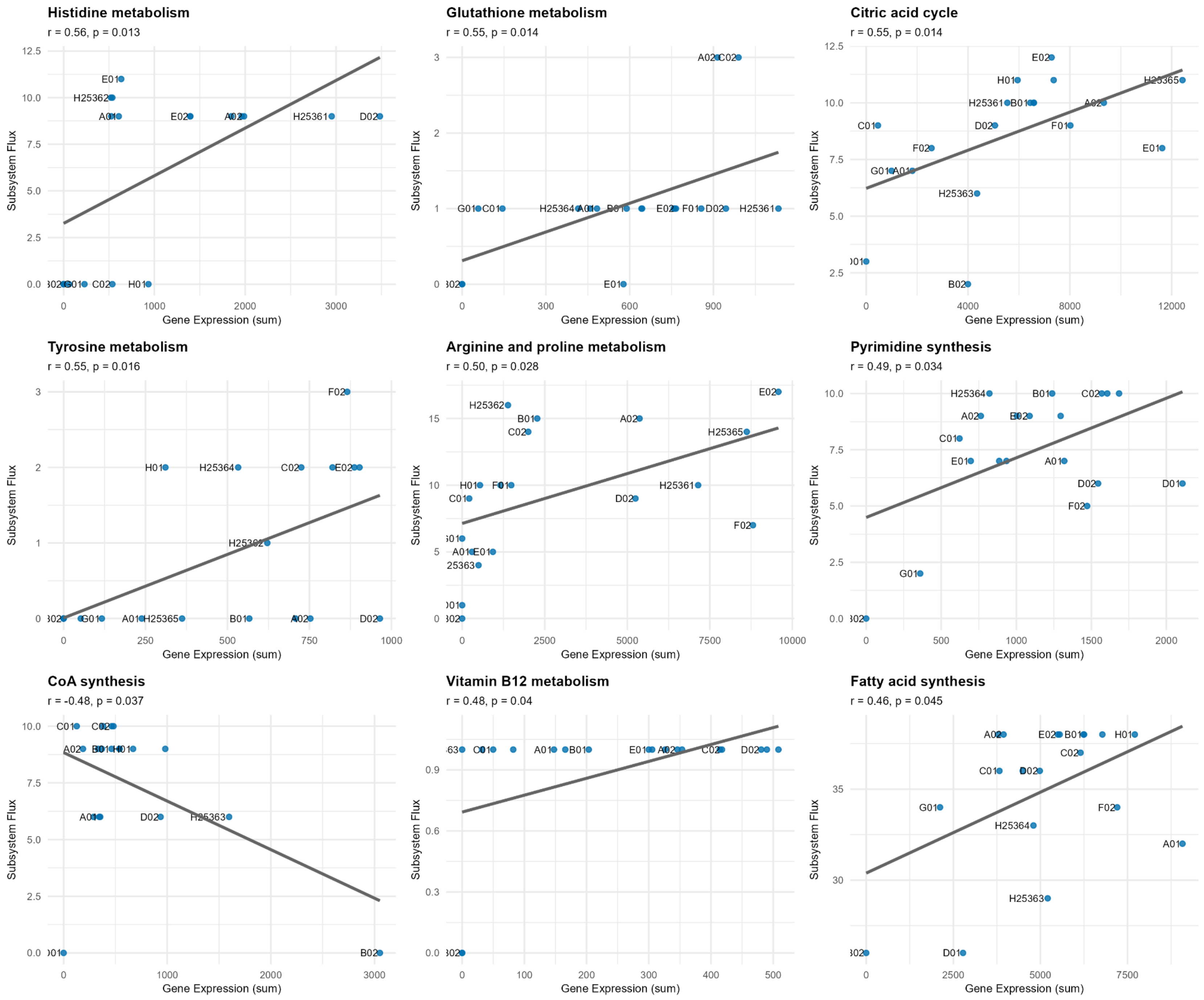
Histidine metabolism
r = 0.56, p = 0.013
Glutathione metabolism
r = 0.55, p = 0.014
Citric acid cycle
r = 0.55, p = 0.014
Tyrosine metabolism
r = 0.55, p = 0.016
Arginine and proline metabolism
r = 0.50, p = 0.028
Pyrimidine synthesis
r = 0.49, p = 0.034
CoA synthesis
r = -0.48, p = 0.037
Vitamin B12 metabolism
r = 0.48, p = 0.04
Fatty acid synthesis
r = 0.46, p = 0.045
Subsystem Flux
Gene Expression (sum)

Supplementary Figure S4. Correlation of summed transcript abundance and predicted flux for nine key metabolic subsystems in *E. coli* UTI89. For each subsystem, patient-specific transcript counts (mapped to both the UTI89 genome and its context-specific metabolic model) are summed and plotted against the model's predicted flux, with each point labeled by its sample ID.

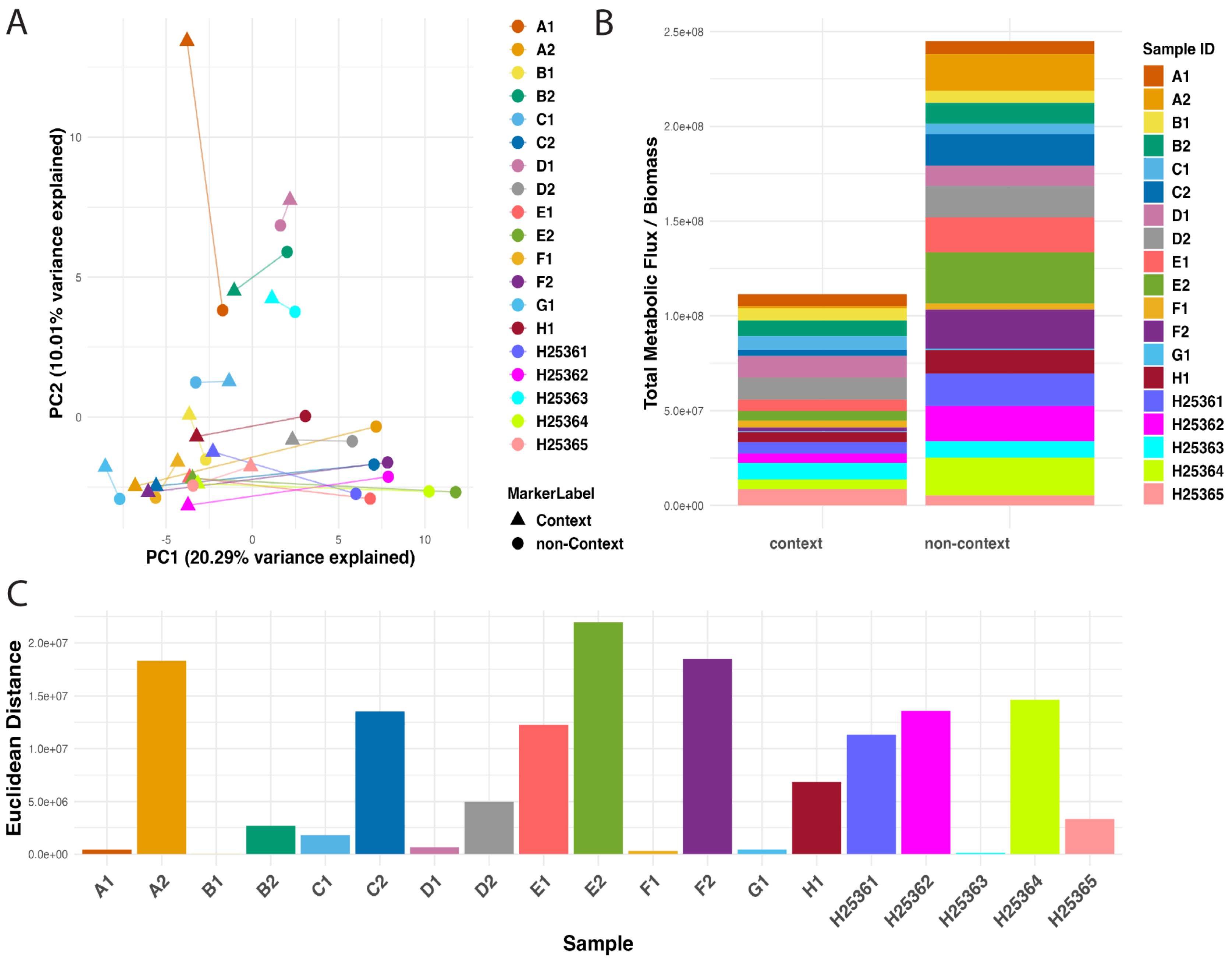
A
PC2 (10.01% variance explained)
PC1 (20.29% variance explained)
10
5
0
-5
0
5
10
A1
A2
B1
B2
C1
C2
D1
D2
E1
E2
F1
F2
G1
H1
H25361
H25362
H25363
H25364
H25365
MarkerLabel
Context
non-Context
B
Total Metabolic Flux / Biomass
2.5e+08
2.0e+08
1.5e+08
1.0e+08
5.0e+07
0.0e+00
context
non-context
Sample ID
C
Euclidean Distance
2.0e+07
1.5e+07
1.0e+07
5.0e+06
0.0e+00
Sample

Supplementary Figure S5: Comparative Metabolic Flux inContext vs Non-ContextMicrobiome Models

(A) PCA plot showing variation in metabolic flux distributions across patient samples by modeling approach. *(B)* Total normalized metabolic flux between context-specific and non-context-specific models. (C) Euclidean distances between the two model types per sample. Larger distances indicate greater methodological influence.

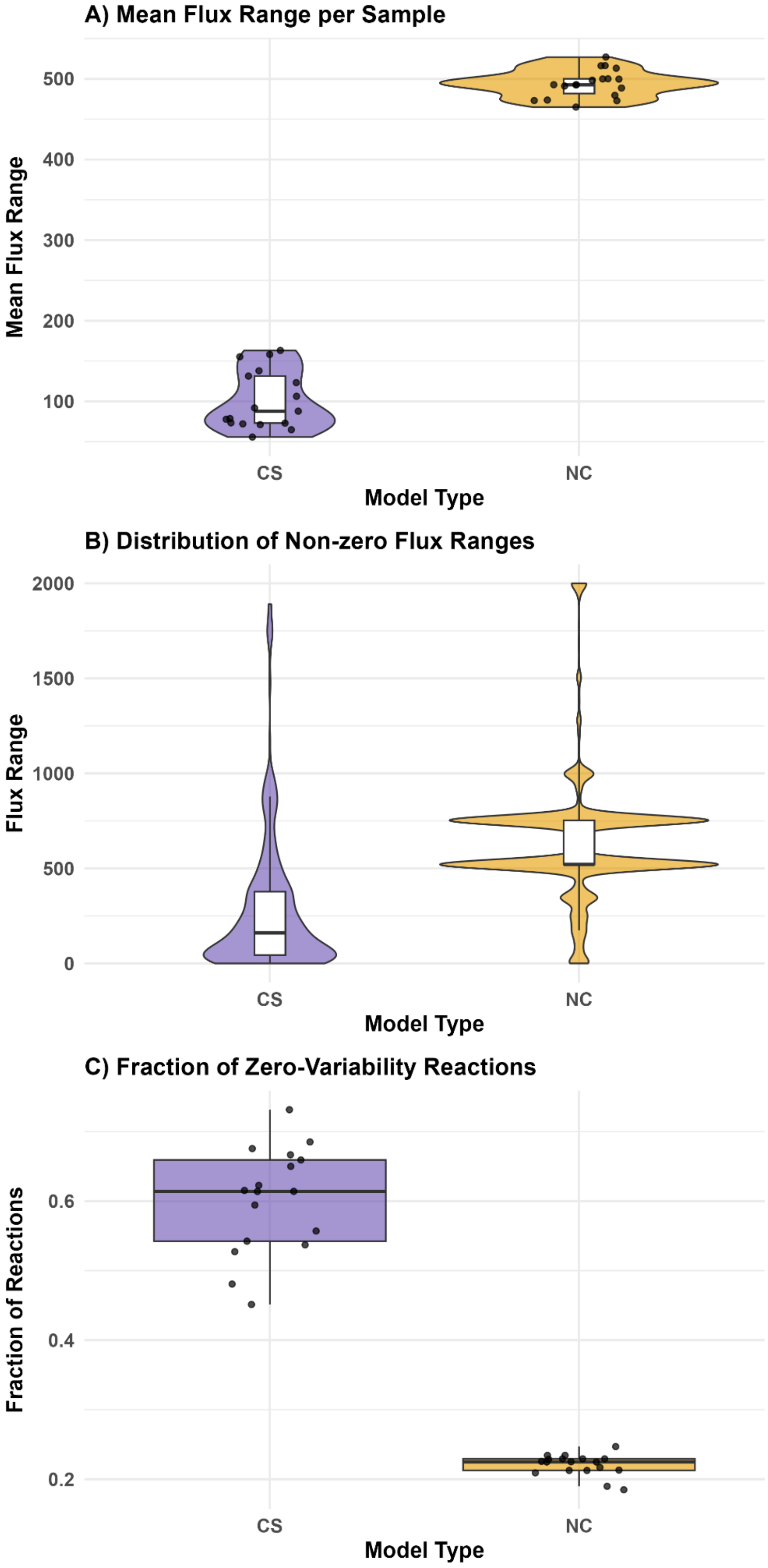

A) Mean Flux Range per Sample
Mean Flux Range
500
400
300
200
100
CS
NC
Model Type
B) Distribution of Non-zero Flux Ranges
Flux Range
2000
1500
1000
500
0
CS
NC
Model Type
C) Fraction of Zero-Variability Reactions
Fraction of Reactions
0.6
0.4
0.2
CS
NC
Model Type

Supplementary Figure S6: Flux Variability Analysis comparing Non-Context-Specific and Context-Specific metabolic models across patient samples. (A) The fraction of reactions with zero flux variability per sample is significantly higher in CS models, indicating tighter constraints on reaction fluxes. (B) Mean flux range per sample is reduced in CS models, showing overall lower variability of metabolic fluxes when constrained by patient-specific transcriptomics. (C) Distribution of non-zero flux ranges aggregated across all samples demonstrates a shift toward narrower flux variability in CS models.
